# Reasoning, Learning, and Creativity: Frontal Lobe Function and Human Decision-Making

**DOI:** 10.1371/journal.pbio.1001293

**Published:** 2012-03-27

**Authors:** Anne Collins, Etienne Koechlin

**Affiliations:** 1 Département d'Etudes Cognitives, Ecole Normale Superieure, Paris, France; 2 Department of Cognitive, Linguistic and Psychological Sciences, Brown University, Providence, Rhode Island, United States of America; 3 Université Pierre et Marie Curie, Paris, France; 4 Laboratoire de Neurosciences Cognitives, Institut National de la Santé et de la Recherche Médicale, Paris, France; California Institute of Technology, United States of America

## Abstract

The frontal lobes subserve decision-making and executive control—that is, the selection and coordination of goal-directed behaviors. Current models of frontal executive function, however, do not explain human decision-making in everyday environments featuring uncertain, changing, and especially open-ended situations. Here, we propose a computational model of human executive function that clarifies this issue. Using behavioral experiments, we show that unlike others, the proposed model predicts human decisions and their variations across individuals in naturalistic situations. The model reveals that for driving action, the human frontal function monitors up to three/four concurrent behavioral strategies and infers online their ability to predict action outcomes: whenever one appears more reliable than unreliable, this strategy is chosen to guide the selection and learning of actions that maximize rewards. Otherwise, a new behavioral strategy is tentatively formed, partly from those stored in long-term memory, then probed, and if competitive confirmed to subsequently drive action. Thus, the human executive function has a monitoring capacity limited to three or four behavioral strategies. This limitation is compensated by the binary structure of executive control that in ambiguous and unknown situations promotes the exploration and creation of new behavioral strategies. The results support a model of human frontal function that integrates reasoning, learning, and creative abilities in the service of decision-making and adaptive behavior.

## Abstract 2

Computational modeling and behavioral experimentation suggest that human frontal lobe function is capable of monitoring three or four concurrent behavioral strategies in order to select the most suitable one during decision-making.

## Author Summary

Reasoning, learning, and creativity are hallmarks of human intelligence. These abilities involve the frontal lobe of the brain, but it remains unclear how the frontal lobes function in uncertain or open-ended situations. We propose here a computational model of human executive function that integrates multiple processes during decision-making, such as expectedness of uncertainty, task switching, and reinforcement learning. The model was tested in behavioral experiments and accounts for human decisions and their variations across individuals. The model reveals that executive function is capable of monitoring three or four concurrent behavioral strategies and infers online strategies' ability to predict action outcomes. If one strategy appears to reliably predict action outcomes, then it is chosen and possibly adjusted; otherwise a new strategy is tentatively formed, probed, and chosen instead. Thus, human frontal function has a monitoring capacity limited to three or four behavioral strategies. The results support a model of frontal executive function that explains the role and limitations of human reasoning, learning, and creative abilities in decision-making and adaptive behavior.

## Introduction

The ability to adapt to uncertain, changing, and open-ended environments is a hallmark of human intelligence. In such natural situations, decision-making involves exploring, adjusting, and exploiting multiple behavioral strategies (i.e., flexible mappings associating stimuli, actions, and expected outcomes [1]–[4]). This faculty engages the frontal lobe function that manages *task sets*—that is, active representations of behavioral strategies stored in long-term memory—for driving action [5]–[10]. According to reinforcement learning (RL) models [11],[12], the task set driving ongoing behavior (referred to as the *actor*) is adjusted according to outcome values for maximizing action utility. Uncertainty monitoring (UM) models [13],[14] further indicate that the frontal executive function infers online the actor *reliability*—that is, its ability to infer action outcomes—for resetting the actor whenever it becomes unreliable. Moreover, models combining RL and UM suggest that given a *fixed* collection of concurrent task sets, the frontal function monitors in parallel their *relative* reliability for adjusting and choosing the most reliable actor [15]–[17].

These models, however, do not explain how the frontal executive function controls an expanding repertoire of behavioral strategies for acting in changing and open-ended environments: that is, how this function decides to create new strategies rather than simply adjusting and switching between previously learned ones. For example, imagine you want to sell lottery tickets to people. After a few trials, you have certainly learned a strategy that appears to be successful for selling your tickets, but your strategy then starts to fail with the next person. You then decide to switch to a new strategy. After adjusting to the new strategy and several successful trials, the new strategy fails again. You may then decide to return to your first strategy or test an entirely new one, and so on. After many trials you have probably learned many different strategies and switch across them and possibly continue to invent new ones. Moreover, among this large collection of behavioral strategies, you may have further learned that several are appropriate with young people, others with older people, some with those wearing hats, others with those holding an umbrella, and so on. How do we learn and manage such an expanding collection of behavioral strategies and decide to create new ones rather than simply adjusting and switching between previously learned ones, possibly according to environmental cues? More formally, little is known about how the frontal executive function continuously arbitrates between (1) adjusting and staying with the current actor set, (2) switching to other learned task sets, and (3) *creating* new task sets for driving action. This issue raises a computational problem that statistical learning models based on Dirichlet process mixtures address [18]–[20]. However, it remains unclear how the frontal executive function may implement such statistical models, because they critically rely on *off-line* Bayesian inferences operating on expanding collections of sets that rapidly become computationally intractable [21]. Thus, a fundamental issue is to understand how with limited monitoring resources the human executive function controls online the creation of new behavioral strategies and *consequently* manages an expanding collection of behavioral strategies for driving action.

To clarify this issue, we proposed a computational model of the frontal executive function that controls the creation, learning, storage, retrieval, and selection of behavioral strategies driving action. The model constitutes a biologically plausible, online algorithm. The algorithm approximates Dirichlet process mixtures [19] by combining reinforcement learning, limited Bayesian inferences, and hypothesis testing for arbitrating between adjusting, switching, and creating actor task sets. Consistent with the capacity limit of human working memory [22]–[24], the model assumes that the frontal executive function forms and monitors in parallel only a *limited* number of concurrent task sets: the executive function monitors only a small part of behavioral strategies stored in long-term memory [22],[23],[25]. As previously suggested [15]–[17], task set reliability is inferred online for choosing the actor sets that drive behavior and adjust to external contingencies. The key assumption is that new task sets are tentatively created and probed as actors whenever no current task sets appear to be reliable. Such probe actors are partly formed by recombining the strategies stored in long-term memory according to external cues [22],[23],[25]. Probe task sets adjust to external contingencies, but may be subsequently discarded when they ultimately appear unnecessary. In the converse case, task set collection is updated with probe task sets: in case the monitoring capacity would be reached, the least recently used task sets are discarded but the associated strategies remain stored in long-term memory. Thus, with limited computing resources, the executive function manages an expanding repertoire of behavioral strategies and controls the selection, learning, retrieval, and creation of behavioral strategies that drive action.

We provided a proper computational formulation of this model, named the PROBE model. We tested the model predictions in behavioral experiments inspired from the standard neuropsychological test of frontal executive function, namely the Wisconsin Card Sorting Test [26],[27]. We compared the PROBE model to alternative models, ruling out successively key model assumptions: the notion of hypothesis-testing on task set creation (MAX model), that of task set creation (FORGET model, which encompasses existing models), and the notion of task set monitoring (RL models). We found that unlike these alternative models, the PROBE model predicts human decisions and their variations across individuals. Moreover, the PROBE model that best fits human data is endowed with a monitoring capacity of three or four task sets.

## Results

### Standard Model Assumptions

We assumed that task sets represent behavioral strategies stored in long-term memory. Each behavioral strategy consists of a *selective* mapping encoding stimulus-response associations, a *predictive* mapping encoding expected action outcomes given stimuli [13]–[15], and a *contextual* mapping encoding external cues predicting task set reliability (see Figure S1 and Materials and Methods).

The executive function builds and monitors at most *N* task sets, a bound reflecting the capacity limit of human working memory [22]–[24]. Consistent with previous studies [13]–[15], task set reliability is evaluated online through forward Bayesian inference: the reliability is inferred before acting according to the perceived volatility of external contingencies [14] and the occurrence of external cues (given contextual mappings) for choosing the actor driving immediate behavior (see below). The actor selective mapping then determines the response to stimulus using a softmax policy (inverse temperature *β* and noise *ε*) [11],[15],[28]. Thus, we assumed that in agreement with previous studies (e.g., [6],[29],[30]), selection happens at the level of task sets first, then at the level of actions within task sets.

After action, selective mappings then adjust according to outcome values through standard reinforcement learning (learning rate *α_s_*) [11],[31], while predictive mappings update outcome predictions [13]. Task set reliability is also updated according to action outcomes (given predictive mappings) and serves to adjust contextual mappings through a classical stochastic gradient descent (contextual learning rate *α_c_*). Contextual mappings thus learn the external cues predicting actual reliability (referred to as contextual cues for clarity).

### PROBE Model

The PROBE model assumes that external contingencies are variable and generated from distinct external states. External states are potentially infinite and not directly observable, thereby reflecting variable, uncertain, and open-ended environments. The PROBE model then builds task sets as instances of external hidden states for appropriately driving behavior according to inferred external states. The reliability of every task set then measures the likelihood that the task set matches current external states given all observable events (contextual cues and the history of action outcomes). For inferring online the opportunity to create new task sets, the PROBE model evaluates task set “absolute” reliability; by concurrently monitoring the reliability of “random behavior,” the PROBE model estimates online the likelihood that no task sets match current external states and, consequently, the reliability of every task set conditional upon the history of action outcomes (and contextual cues) but not upon the collection of current task sets (see Materials and Methods).

Consequently, when a task set appears to be *reliable* (i.e., more likely reliable than unreliable), it becomes the actor (i.e., the exclusive action selector) because no others meet this criterion. Conversely, whenever no task sets appear to be reliable, a new task set is created and probed as the actor. This actor initially consists of new selective/predictive mappings, which are formed from mixing selective/predictive mappings stored in long-term memory and weighted according to contextual cues (given contextual mappings) [22],[23],[25]. The mixture is prone to noise scaled by parameter *η* named *recollection entropy* (0≤*η*≤1). Endowed with prior reliability minimizing prior information [32], the probe actor is initially unreliable, but its selective/predictive mapping adjusts to external contingencies: when it becomes reliable, while the other task set remains unreliable, task set creation is “confirmed”; task set collection is updated by possibly discarding the least recent actor set in case the capacity limit would be reached. When conversely another task set becomes reliable before the probe actor, the latter is discarded and the former becomes the actor. Thus, the PROBE model is an online, forward approximation of Dirichlet process mixtures [19] based on hypothesis testing on task set creation (i.e., on the critical no-parametric component of Dirichlet processes) (see Text S1).

In the PROBE model, unselected task sets are inferred as being *unreliable* (i.e., unrelated to current external states). The PROBE model therefore assumes that unlike multiple actor models [15]–[17], no learning occurs in selective and predictive mappings within unselected task sets. Thus, only selective/predictive mappings of actor task sets are adjusted according to action outcomes. This assumption is consistent with empirical evidence that in task switching, task set selection inhibits internal mappings of unselected task sets (e.g., [6],[29],[30]).

Overall, the PROBE model has six free parameters. Standard free parameters are: inverse temperature *β* scaling greediness in action selection, noise *ε* scaling lapses probability in action selection, and learning rates *α_s_* and *α_c_* scaling updating rates of selective and contextual mappings. Additionally, we treated bounds *N* and recollection entropy *η* as free parameters for investigating multiple theoretical schemes. We also considered two additional free parameters capturing possible human biases (Materials and Methods): *context-sensitivity bias δ*>0 increasing transiently the perceived volatility of external contingencies (i.e., the tendency to switch actors whenever, besides stimuli, additional external cues change between two successive trials) and *confirmation bias θ* enhancing prior reliability of newly formed task sets, thereby restraining their immediate disengagement.

### Alternative Models

The MAX model is identical to the PROBE model, except that it removes the notion of hypothesis testing for creating task sets. New task sets are created for acting only when no task sets appear more reliable than “random behavior” (i.e., when it becomes more likely that no task sets match current external states) (see Text S1). Endowed with prior reliability corresponding to random behavior, new task sets therefore appear initially as the most reliable ones, so that task set creation is automatically confirmed. Thus, the most reliable task set is the actor, provided that it remains more reliable than random behavior. The MAX model creates new task sets *only when* no current task sets are more reliable than chance, whereas the PROBE creates new task sets *once* no current task sets appear to be reliable. Conversely, the MAX model keeps new task sets in the monitoring buffer when there are no more actors, whereas the PROBE model keeps them *provided that* they have been reliable. The MAX model corresponds to the one-particle filtering approximation of Dirichlet process mixtures [21]. Otherwise, the MAX and PROBE models are identical and have the same free parameters.

The FORGET model further removes the notion of task set creation (Text S1). The actor is chosen using a softmax policy (inverse temperature *β′*) for possibly recycling task sets. Concomitantly, the strategies associated with unused task sets decay into the random strategy (decay rate *ϕ*, 0<*ϕ<1*) [33],[34], so that unused task sets may be recycled as “new” task sets. Thus, the collection of task sets is fixed and corresponds to monitoring capacity *N*. As external states are potentially infinite, task set reliability therefore represents relative evidence across distinct behavioral strategies rather than external states. The FORGET model therefore assumes that as in multiple actor models [15]–[17] selective/predictive mappings are adjusted concurrently in every task set in proportion to task set reliability. For consistency with both the PROBE and MAX models, we also tested the FORGET model with the assumption that learning occurs only for actor task sets. In the present study, the two assumptions actually yield to virtually the same predictions, so we ignore the distinction henceforth.

The FORGET model encompasses existing models: basic RL models when bound *N* = 1 [11],[12], UM models when *N* = 2 and decay rate *ϕ* is large relative to external volatility [13],[14], and finally, multiple actor models combining RL and UM when *N*>1 and ϕ = 0 [15]–[17]. The FORGET model has the same free parameters as the MAX and PROBE models, except that decay rate *ϕ* and inverse temperature *β′* replace recollection entropy and confirmation bias, respectively.

### Human Decisions With No Contextual Cues

We conducted the first experiment with 22 participants who responded to successive visual stimuli (three possible digits) by pressing one among four response buttons (see Figure S2A and Materials and Methods). For each stimulus, one response led to a positive outcome with a probability of 90% (audiovisual feedbacks associated with extra monetary payoff), while the others led to a positive outcome with a probability of 10% only. Unbeknownst to the participants, the mapping between stimuli and best responses shifted after an unpredictable number of trials, ranging from 36 to 54. No cues predicted such changes. We refer to a series of trials occurring between two successive changes as an *episode*. Without being instructed, moreover, participants performed two distinct sessions. In the *open* session, every episode corresponded to new stimulus response mappings, whereas in the *recurrent* session, only three mappings reoccurred unpredictably; every episode corresponded to one among these three mappings, so that participants could reuse what they previously learned.

**Figure 1:**
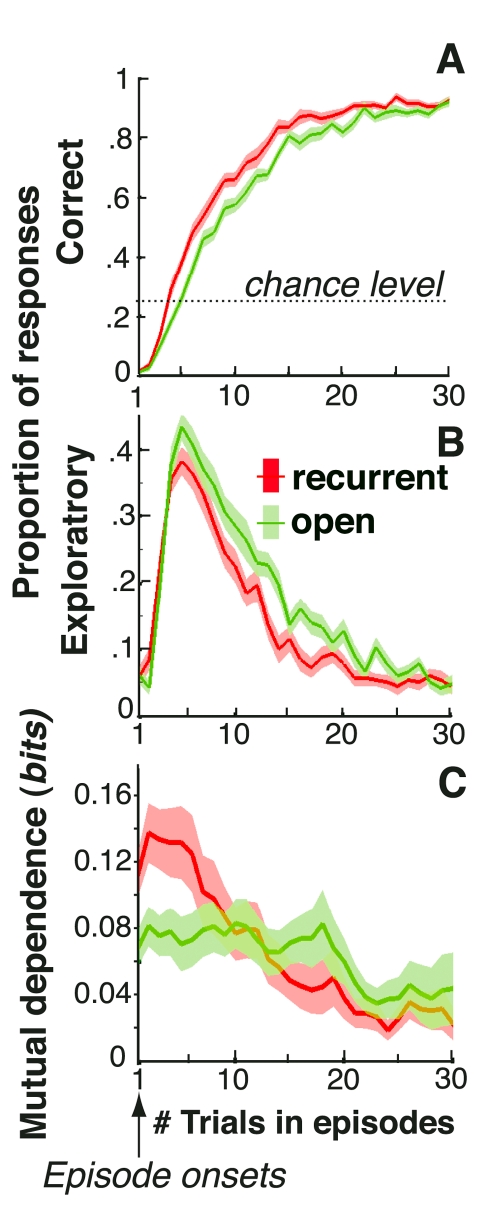
Human decisions with no contextual cues. Participants' performances in recurrent (red) and open (green) episodes plotted against the number of trials following episode onsets. Shaded areas are S.E.M. across participants. (A) Correct response rates. (B) Exploratory response rates. (C) Mutual dependence (i.e., mutual information) of two successive correct decisions averaged over five-trial sliding bins (see Text S1).

Following episode changes, participants then produced perseverative responses (best responses in the preceding episode), correct responses (best responses in the ongoing episode), or exploratory responses (neither perseverative nor correct). In both conditions, correct response rates increased from ∼2% at episode onsets to ∼90% about 30 trials later (chance level: 25%). Exploratory response rates increased from ∼5% at episode onsets, peaked at ∼40% about three or four trials later, and then gradually returned to ∼5% (chance level: 50%) (Figure 1A,B). Thus, in all episodes, participants maximized pay-offs by learning the associations between stimuli and correct responses. Critically, correct responses increased and exploratory responses vanished faster in the recurrent than open episodes (both *t*s>3.4, *p*s<0.005). Thus, in recurrent episodes, participants retrieved the appropriate associations they had previously learned, although in the meantime they learned incongruent associations.

Moreover, we found that in the first trials of recurrent episodes, a positive feedback caused the production of a correct response in the next trial even when the two successive stimuli differed. Indeed, the mutual dependence between two successive correct decisions strongly increased in the first trials of recurrent compared to open episodes (*t* = 2.8, *p* = 0.012, Figure 1C and Text S1). In the following trials, by contrast, this mutual dependence remained weak, approximately constant, and similar in both recurrent and open episodes (*t*<1). This finding shows that in the first trials of recurrent episodes, participants used feedbacks to retrieve the appropriate stimulus-response mapping rather than recollecting each stimulus-response association separately. Consequently, participants built and stored multiple stimulus-response mappings and monitored action outcomes for retrieving previously learned mappings or learning new ones. This finding further confirms that the improved performance in the recurrent compared to open condition could not arise from faster learning rates in recurrent than open episodes. Indeed, learning rates are presumed to increase with uncertainty [35],[36] and should instead be faster in open episodes that feature increased uncertainty.

**Figure 2:**
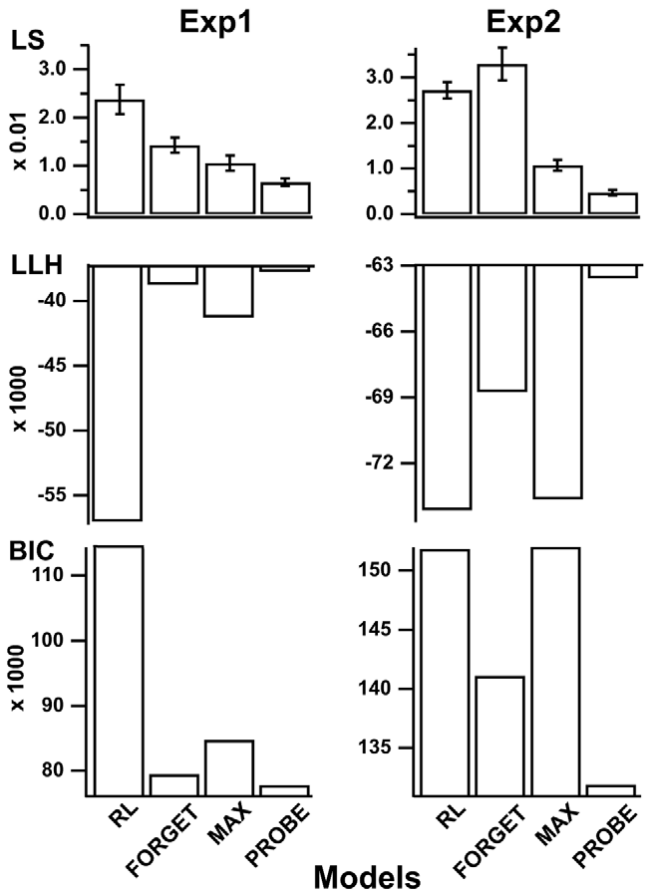
Comparison of model fits. Models were fitted using the standard maximum log-likelihood (LLH) and least squares (LS) methods. Histograms show the LS and LLH as well as the Bayesian information criterion (BIC) obtained for each model. The LLH method maximizes the predicted (log-)likelihood of observing actual participants' responses. The LS method minimizes the square difference between observed frequencies and predicted probabilities of correct responses. The Bayesian information criterion (BIC) alters LLH values according to model complexity favoring models with less free parameters (Text S1). Larger LLH, lower LS, and lower BIC values correspond to better fits. Left, first experiment with no contextual cues. Parameters that cannot be estimated (i.e., contextual learning rate *α_c_* and context-sensitivity bias *δ*) were removed from the fitting. RL, basic reinforcement learning model including a single task-set learning stimulus-response association (free parameters: inverse temperature *β*, noise *ε*, learning rate *α_s_*). Right, second experiment with contextual cues. RL, pure reinforcement learning model learning a mixture of stimulus-response and stimulus-cue-response associations (free parameters: inverse temperature *β*, *β′* noise *ε*, learning rates *α_s_* and *α_c_*, and mixture rate *ω*; see Text S1). Note that in both experiments the PROBE model was the best fitting model for every fitting criterion (LS, all *F*s>3.8, *p*<0.001).

**Figure 3:**
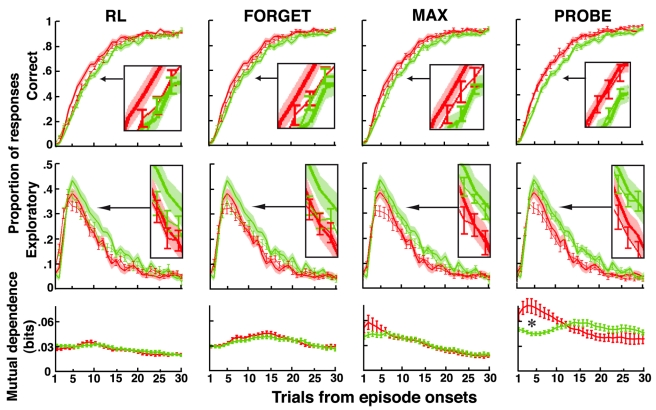
Predicted versus observed decisions with no contextual cues. Correct and exploratory response rates as well as mutual dependences of successive correct decisions in recurrent (red) and open (green) episodes plotted against the number of trials following episode onsets. Lines ± error bars (mean ± S.E.M.): performances predicted by fitted RL, FORGET, MAX, and PROBE models. RL, reinforcement learning model including a single actor learning stimulus-response associations (details in Figure 2, legend). Correct and exploratory response rates were computed in every trial according to the actual history of participants' responses. Mutual dependence of successive correct decisions predicted by each fitted model was computed as the mutual information between two successive correct responses produced by the model independently of actual participants' responses (one simulation for each participant). Stars show significant differences at *p*<0.05 (mutual dependences on the first eight trials between recurrent and open episodes. *t* tests, RL & FORGET, all *t*s<1. MAX, all *t*s<2, *p*s>0.06; PROBE, all *t*s>3.2, *p*s<0.004). Lines ± shaded areas (mean+S.E.M.): human performances (data from Figure 1). Insets magnify the plots for Trials 7, 8, and 9. See Table S1 for fitted model parameters. See Text S1 for the discrepancy observed in Trial 5 between participants' exploratory responses and model predictions (section “Comments on Model Fits”).

To understand this human ability, we computed for every participant the models' parameters that best predict his or her choice in every trial given his or her previous responses (Figure 2, legend). As expected, the three models fit participants' responses significantly better than a basic RL model adjusting for a single actor, even when penalizing for increased model complexity (Figure 2, left). However, neither the fitted FORGET, MAX, nor RL model accounted for the differential performances observed between the recurrent and open episodes (Figure 3). Indeed, the best fitting FORGET model was obtained with bound *N* = 2 (*M* = 2.2; S.E.M. = 0.16) and large decay rate *ϕ* (*M* = 14%, S.E.M. = 0.9%) relative to the volatility of external contingencies (3%). This model therefore reduces to a standard UM model [13],[14] that monitors only the actor reliability relative to chance with no ability to retrieve previously learned mappings. Similarly, the best fitting MAX model was obtained with bound *N* = 1 (*M* = 1.4; S.E.M. = 0.14). This model again monitors only the actor reliability relative to chance; previously learned mappings are retrieved only by creating new task sets from strategies stored in long-term memory with no guidance from action outcomes. The model therefore fails to account for the increased mutual dependence of successive decisions made in the first trials of recurrent episodes (Figure 3).

By contrast, the PROBE model predicts participants' responses and their successive dependence in both recurrent and open episodes (Figure 3). Consistently, the PROBE model fits participants' responses significantly better than the other models (Figure 2, left). The best fitting PROBE model was obtained with bound *N* = 3 (*M* = 3.3; S.E.M. = 0.3); in recurrent episodes, previously learned mappings are retrieved by selecting the appropriate task sets according to action outcomes; this explains the increased dependence of successive decisions made in the first episode trials. In open episodes, by contrast, new task sets are created for driving behavior and learning the new mappings, when facing new external contingencies that cannot be reliably predicted.

We then tested the hypothesis underlying the PROBE model that action selection involves a two-stage process: first choosing the actor task set and then selecting actions within the actor task set. For that purpose, we considered a variant of the PROBE model that rules out this hypothesis: actions are directly selected by marginalizing over task sets on the basis of task sets' reliability. In this variant, consistently, concurrent learning occurs for every task set in proportion to task set reliability. Again, the best fitting variant was obtained with monitoring bound *N* = 1, so that the variant becomes equivalent to the best fitting FORGET and MAX models and similarly fails to account for the differential performances observed between the recurrent and open episodes. Thus, the data support the PROBE model assumption that action selection is based on first choosing the actor task set according to task set reliability and then selecting actions according to the actor selective model.

Finally, we compared the PROBE model parameters that best fit participants' responses (see Table S1) to those optimizing PROBE model performance in this protocol. Using computer simulations, the optimal PROBE model parameters were computed as those maximizing the proportion of correct responses produced by the model over both sessions irrespective of participants' data (optimal PROBE model performance, 80%; participants' performance ± S.E.M., 77%±0.6%). As expected, optimal bound *N* was equal to 3, and optimal recollection entropy *η* was equal to 1 (the maximal value); because the optimal model is able to monitor the exact number of recurrent mappings in the recurrent condition, the recollection of behavioral strategies from long-term memory becomes useless. As mentioned above, best fitting bound *N* averaged across participants was similar to the optimal value (*M* = 3.3; S.E.M. = 0.3). Compared to the optimal PROBE model, however, participants exhibited lower recollection entropy *η* (*η_best-fitting_* ± S.E.M. = 0.72±0.07) and positive confirmation bias (*θ_optimal_* = 0; *θ_best-fitting_* = 0.74±0.12). This indicates that participants retrieved learned behavioral strategies by relying more on long-term memory recollection than optimally on working memory retrieval (monitoring buffer). This is consistent with the fact that in several participants, monitoring bound *N*s were lower than the number of recurrent mappings.

Regarding action selection within task sets, optimal inverse temperature was large and equal to 30 and optimal noise ε equal to 0. As expected, the optimal model behavior is greedy and most often selects best rewarding responses. Interestingly, participants were as greedy as the optimal model behavior with similar best-fitting inverse temperature *β* (32±2) and virtually zero noise *ε* (0.01±0.003). Optimal and best fitting learning rates of selective mappings *α_s_* were also similar (*α_s(optimal)_* = 0.4; *α_s(best-fitting)_* = 0.41±0.03), indicating that participants efficiently stored behavioral strategies in long-term memory.

### Human Decisions with Contextual Cues

In a second experiment, we examined whether in the presence of contextual cues predicting current external contingencies the PROBE model remains the best predictor of participants' decisions. Forty-nine additional participants first carried out the same *recurrent* session as described above, except that unbeknownst to them, stimulus colors informed current mappings between stimuli and best responses. These contextual cues therefore switched at episode onsets and sometimes within episodes, because the same mapping could be associated with distinct color cues (see Figure S2B and Materials and Methods).

**Figure 4:**
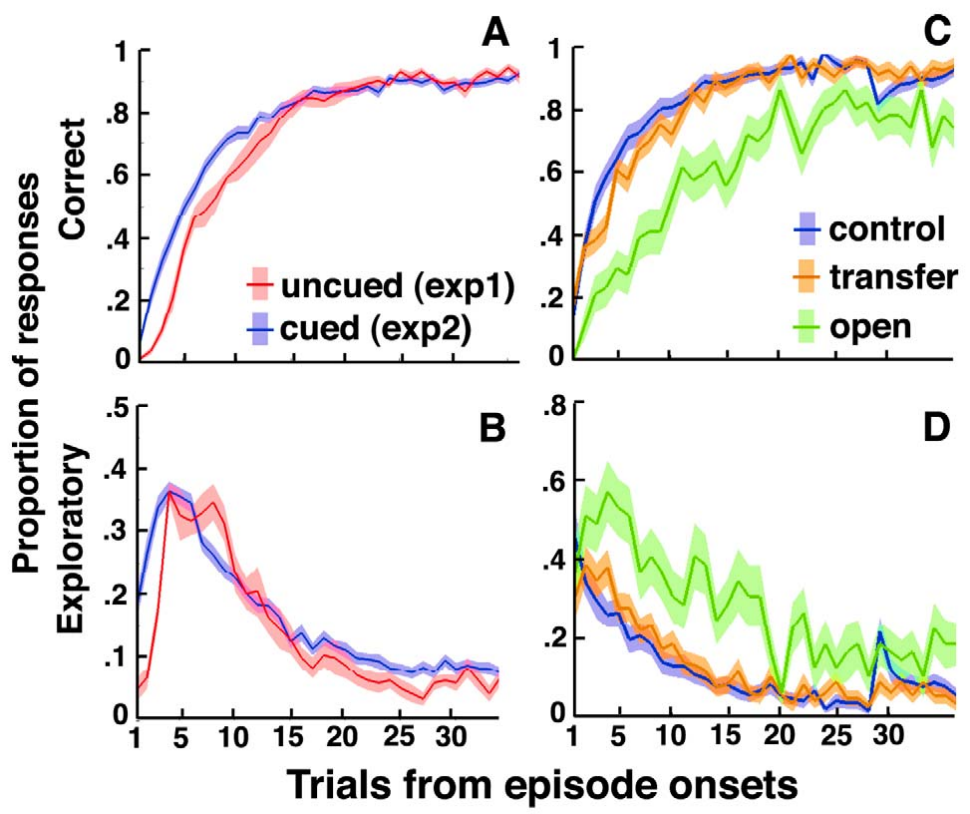
Human decisions with contextual cues. Participants' performances are plotted against the number of trials following episode onsets. Shaded areas are S.E.M. across participants. (A and B) Correct and exploratory response rates in uncued (red) and cued (blue) recurrent episodes. Uncued recurrent episodes are from Experiment 1 for participants who performed the recurrent session before the open session (half of participants). Cued recurrent episodes correspond to the first session of the second experiment. (C and D) Correct and exploratory response rates in control (blue), transfer (orange), and open (green) episodes (second experiment, second session). In control episodes, the drop of correct response rates and the peak of exploratory response rates visible on Trial 29 corresponded to contextual cue changes while external contingencies remained unchanged (see Figure S3).

In these cued recurrent episodes, participants roughly behaved as in previous, uncued recurrent episodes (Figure 4A,B). Following episode changes, however, correct responses increased and exploratory responses vanished earlier in cued than in uncued episodes. These effects were even observed in the first episode trial before the first (adverse) feedback (both *t*s>4; *p*<0.001), indicating that participants used contextual cues to switch behavior proactively.

Participants then carried out a second session identical to the first one, except that unbeknownst to them, the session intermixed three types of cued episodes: *control* episodes corresponding to cued recurrent episodes encountered in the first session, *transfer* episodes corresponding to such recurrent episodes but associated with new contextual cues, and *open* episodes corresponding to new mappings and contextual cues.

Following episode changes, correct responses increased and exploratory responses vanished similarly in control and transfer episodes (both *t*s<1.5, *p*s>0.13) but faster and earlier in these episodes than in open episodes (all *t*s>4.4, *p*s<0.001, Figure 4C,D). Participants therefore performed without using a single “flat” actor directly learning stimulus-cue-response associations. Indeed, in this case, the performance in transfer episodes would have been similar to the performance in open rather than control episodes.

**Figure 5:**
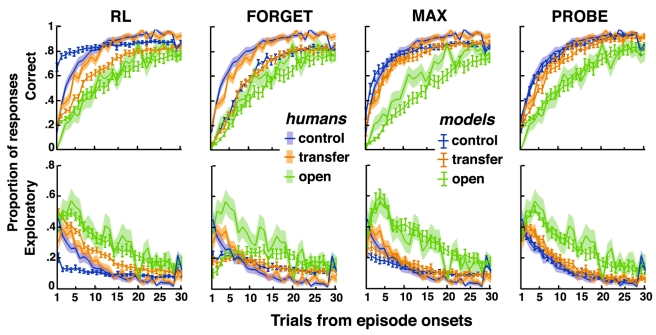
Predicted versus observed decisions with contextual cues. Correct and exploratory response rates in control (blue), transfer (orange), and open (green) episodes plotted against the number of trials following episode onsets. Lines ± error bars (mean ± S.E.M.): performances predicted by fitted RL, FORGET, MAX, and PROBE models in every trial according to the actual history of participants' responses. The RL model includes a single actor learning a mixture of stimulus-response and stimulus-cue-response associations (see Figure 2 legend for details). Lines ±shaded areas (mean+S.E.M.): human performances (data from Figure 4C,D). See Table S1 for fitted model parameters. Note the systematic discrepancies between the predictions from RL, FORGET, and MAX models and human data.

For every participant, as described above, we then computed the models' parameters that best predict the participants' responses. Again, the PROBE model was the best fitting model, even when compared to pure RL models learning mixtures of stimulus-response and stimulus-cue-response associations (Figure 2, right). Unlike the other models, the PROBE model predicts participants' performances in control, transfer, and open episodes (Figure 5). Moreover, the best fitting PROBE model was again obtained with bound *N* = 3 (*M* = 3.2; S.E.M. = 0.3). Other model parameters were also similar to those obtained in the first experiment with no contextual cues (mean ± S.E.M.: recollection entropy *η* = 0.84±0.02; confirmation bias *θ* = 0.71±0.06; inverse temperature *β* = 25±2; noise *ε* = 0.05±0.01), except learning rate *α_s_*, which was lower (0.18±0.1). Compared to the optimal PROBE model, however, participants exhibited lower contextual learning rates (*α_c(optimal)_* = 0.1; *α_c(best- fitting)_* = 0.006±0.002) and large contextual sensitivity bias *δ* (*δ_optimal_* = 0; *δ_best fitting_* = 0.55±0.04). Unlike a participant, the optimal PROBE model perfectly learns the associations between contextual cues and behavioral strategies and uses them to proactively select/retrieve learned behavioral strategies. The discrepancy is consistent with the fact that in the model only color cues were implemented as additional stimulus attributes, whereas participants faced much more contextual information and were not specifically informed about color cues.

### Inter-Individual Variability

Knowing that adaptive behaviors are highly variable and may even qualitatively differ across individuals [37]–[39], we examined inter-individual variability by analyzing separately three groups of participants identified from post-tests. Post-tests assessed participants' ability to recollect the three stimulus-response mappings they learned in recurrent sessions (Text S1). We found that only two-thirds of participants recollected the three mappings (13/22 and 34/49 in the first and second experiment, respectively). We refer to them as *exploiting* participants and to the remaining third as *exploring* participants. Furthermore, in the second experiment, only half of exploiting participants (19/34) recollected the contextual cues associated with learned mappings. We refer to them as *context-exploiting* participants and to the remaining half (15/34) as *outcome-exploiting* participants.

**Figure 6:**
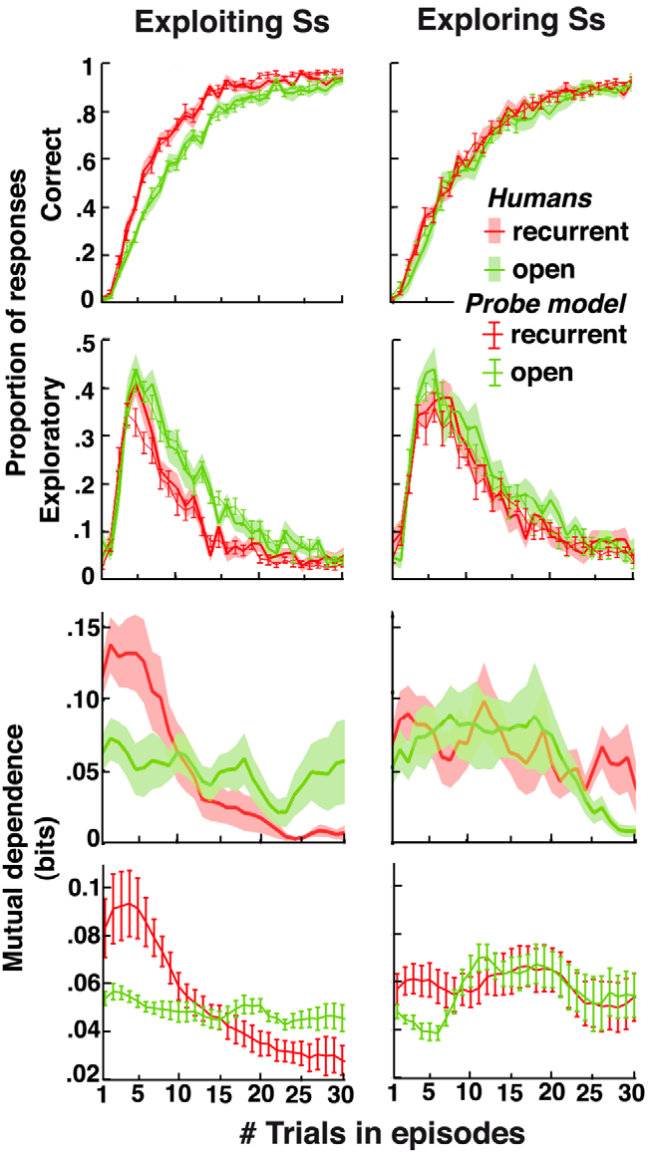
Individual differences in decision-making with no contextual cues. Correct and exploratory response rates as well as mutual dependence of successive correct decisions in recurrent (red) and open (green) episodes plotted against the number of trials following episode onsets (data from Experiment 1). Lines ± shaded areas (mean+S.E.M.): participants' performances. Lines ± error bars (mean ± S.E.M.): predicted performances from the fitted PROBE model. Predicted correct and exploratory response rates were computed in every trial according to the actual history of participants' responses. Predicted mutual dependence of successive correct decisions was computed as the mutual information between two successive correct responses produced by the model independently of actual participants' responses (one simulation for each participant). Left, exploiting participants: Correct responses increased and exploratory responses vanished faster in recurrent than open episodes (Wilcoxon-test, both *z*s>2.8, *p*s<0.005). Right, exploring participants: performances were similar in recurrent and open episodes (correct and exploratory responses: Wilcoxon-test, both *z*s<1.4, *p*s>0.17). See Table S2 for fitted model parameters in each group. See Text S1 for the discrepancy observed in Trial 5 between exploiting participants' exploratory responses and model predictions in recurrent episodes (section “Data Analyses”).

**Figure 7:**
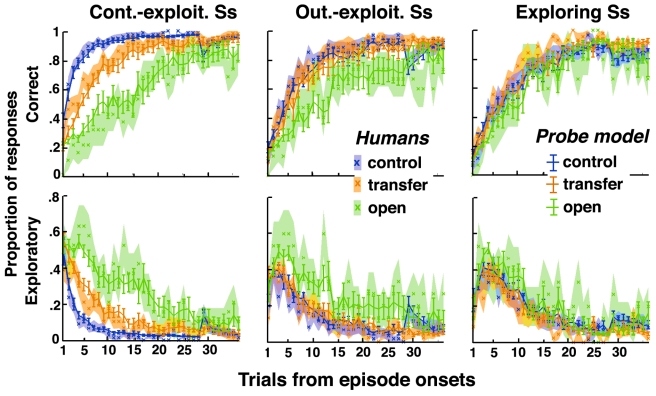
Individual differences in decision-making with contextual cues. Correct and exploratory response rates in control (blue), transfer (orange), and open (green) episodes plotted against the number of trials following episode onsets (data from Experiment 2). Lines ± shaded areas (mean+S.E.M.): participants' performances. Lines ± error bars (mean ± S.E.M.): performances predicted by the fitted PROBE model in every trial according to the actual history of participants' responses. Left, context-exploiting participants: Correct responses increased and exploratory responses vanished faster in control than transfer episodes (Wilcoxon-tests, both *z*s>2.4, *p*s<0.015) and faster in transfer than open episodes (Wilcoxon-tests, both *z*s>3.1, *p*s<0.002). Middle, outcome-exploiting participants: performances were similar in control and transfer episodes (correct and exploratory responses: Wilcoxon-tests, both *z*s<1.4, *p*s>0.15), but correct responses increased and exploratory responses vanished faster in transfer than open episodes (Wilcoxon-tests, both *z*s>2.3, *p*s<0.023). Right, exploring participants: performances were similar in control, transfer, and open episodes (correct and exploratory responses: Friedmann-tests, both χ^2^<5.3, *p*s>0.07). Note that in open episodes, exploring participants adjusted faster than exploiting participants (correct responses: both *t*s>3.0, *p*s<0.004). See Table S2 for fitted model parameters in each group.

Consistently, in both experiments, exploring participants behaved without retrieving previously learned stimulus-response mappings. Unlike exploiting participants, they performed identically across all episodes (Figures 6 and 7). Conversely, only context-exploiting participants adjusted faster in control than transfer episodes (Figure 7), indicating that unlike the others, context-exploiting participants further used contextual cues for retrieving the appropriate mappings. Importantly, these individual differences were unrelated to possible variations in fatigue, attention, or motivation across participants. Indeed, in control and transfer episodes, exploiting participants adjusted faster than exploring participants, but in open episodes, the opposite was observed: exploring participants adjusted faster than exploiting participants (Figure 7, legend). Moreover, no groups ignored contextual cues as shown in Figure S3.

**Figure 8:**
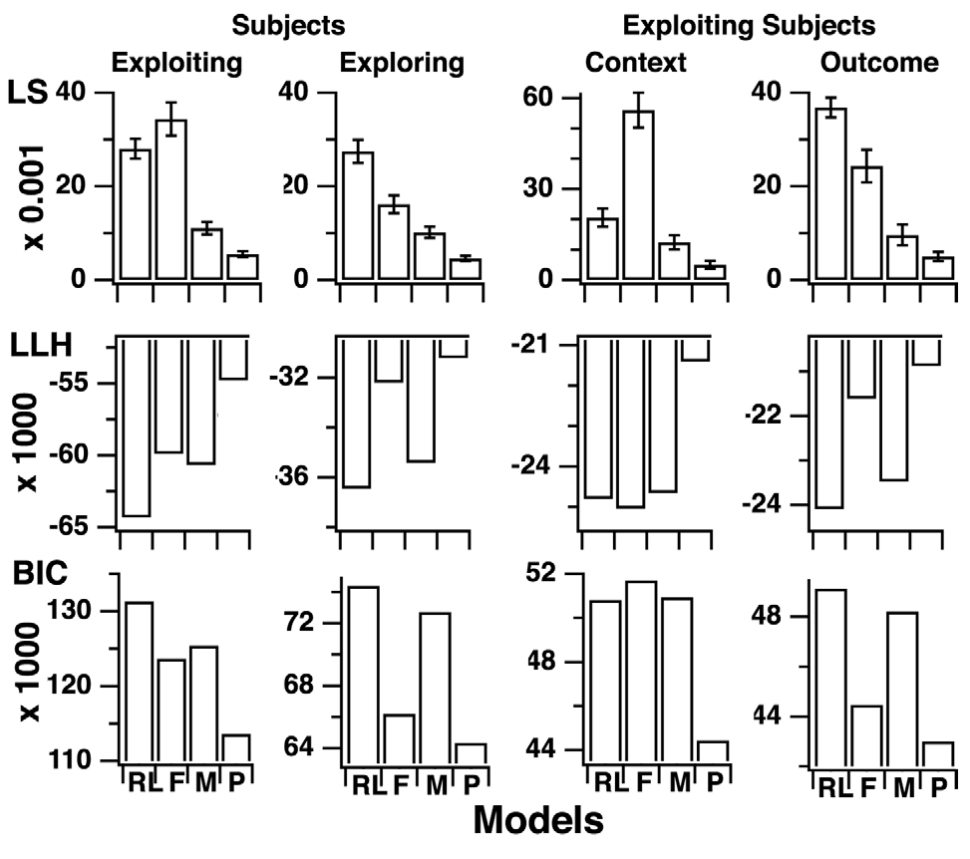
Comparison of model fits according to individual differences. Least square residuals (LS), maximal log-likelihoods (LLH), and Bayesian information criteria (BIC) obtained for each model in exploring versus exploiting participants (left) and in context- versus outcome-exploiting participants (right). RL, reinforcement learning; F, FORGET; M, MAX; P, PROBE model. See details in the Figure 2 legend. Note that in every participants' group, the PROBE model was the best fitting model for every fitting criterion (LS, all *F*s>4.2, *p*s<0.001 in exploiting and exploring groups; Wilcoxon tests in context- and outcome-exploiting groups, all *z*s>2.0, *p*s<0.047).

In every group, the PROBE model precisely predicted participants' behavior (Figures 6 and 7) and strikingly remained the best fitting model (Figure 8). In the best fitting PROBE model, moreover, exploring participants featured only larger *confirmation biases θ* than exploiting participants (*n* = 24 versus 34; Mann-Whitney tests, *p*<0.001; all other parameters, *p*s>0.11). Notably, bounds *N* and recollection entropy *η* were similar between the two groups (*M* ± S.E.M.: *N*_exploring_ = 3.3±0.3; *N*_exploiting_ = 3.0±0.3; *η*_exploring_ = 77%±2%; *η*_exploiting_ = 82%±6%). With only larger confirmation biases, exploring participants appeared simply more prompt than exploiting participants to accept probe actors they created especially when episodes changed. Consistent with their post-test retrieval performances and large recollection entropy, exploring compared to exploiting participants were thus modeled as re-learning from scratch rather than retrieving the stimulus-response mappings they had previously learned.

By contrast, context- compared to outcome-exploiting participants featured only larger *context-sensitivity biases δ*, larger contextual learning rates *α_C_* (*M* = 1.1% versus 0.4%) and slightly lower recollection entropy *η* (*M* = 77%±3% versus 86%±2%) (Mann-Withney tests, all *p*s<0.025; all other parameters, *p*s>0.1). Again, bound *N* was virtually identical in the two groups (*N* = 3.474 versus 3.467, S.E.M.s = 0.4). With larger *context-sensitivity* biases, context- compared to outcome-exploiting participants appeared more prompt to switch behavior whenever contextual cues shifted. In this protocol, this bias along with slightly lower recollection entropy strongly favored the learning of contextual models, because cue changes were most often associated with episode changes. Consistent with their post-test retrieval performances, outcome-exploiting participants were thus modeled as learning more efficiently the associations between contextual cues and stimulus-response mappings.

## Discussion

We found that the best account of human decisions is the PROBE model combining forward Bayesian inference for evaluating task set reliability and choosing the most reliable actor set and hypothesis-testing for possibly creating new task sets when facing ambiguous or unknown situations. Relaxing successively these assumptions, namely hypothesis-testing (MAX model), task set creation (FORGET model), and reliability monitoring (pure RL models), fails to account for human decisions. In contrast to these alternative models, the PROBE model predicts human decisions and its variations across individuals in recurrent or open-ended environments, with variable external contingencies possibly associated with contextual cues.

Critically, the PROBE model estimates the “absolute” reliability of task sets and consequently involves *binary* decision-making for selecting actors, even when multiple task sets are monitored in parallel. Indeed, actor selection is based on a “*satisficing*” criterion based on task set reliability [1]: either a task set appears to be reliable, in which case it becomes the actor, because no other task sets meet this criterion, or no task set appears reliable, in which case a new task set is created and serves as an actor (Materials and Methods). The results thus show that human executive control (i.e., task set selection) involves *binary* decisions based on task set *reliability*. This finding contrasts with action selection within task sets, which in agreement with previous studies [28] involves *multi-valued* decisions based on (soft-) maximizing expected *utility* of actions.

The PROBE model further indicates that in both experiments participants' performances relied on forming and monitoring at most three or four task sets in parallel. This capacity was independent of individual differences in retrieving task sets but might reflect the number of stimulus-response mappings used in recurrent sessions (i.e., three). To examine this possibility, we fit the PROBE model on participants' performances in open sessions only, which include no recurrent episodes. Again, we found that the best fitting PROBE model was obtained with monitoring bound *N* equal to three or four task sets (*M* = 3.4, S.E.M. = 0.5, with no significant differences between open sessions performed first and second: *N* = 2.9±0.6; *N* = 4.0±0.8; Mann-Whitney test, *p*>0.46). This capacity therefore appears to be independent of the protocol structure. Furthermore, we conducted an additional experiment with 30 additional participants that consisted of a recurrent session identical to that used in Experiment 1, except that *four* recurrent mappings between stimuli and correct responses reoccurred pseudo-randomly across episodes. We found that the best fitting monitoring bound *N* was virtually identical to that found in Experiments 1 and 2 (*M* = 3.4, S.E.M. = 0.3) (Figure S4, legend). Thus, monitoring bound *N* was essentially unaltered by the amount of information stored in long-term memory (selective and predictive mappings). In this session, moreover, participants performed as in open episodes (Figure S4), indicating that, on average, participants monitored no more than three task sets. Altogether, the results provide evidence that, on average, the monitoring capacity of human executive function (also referred to as procedural working-memory [23],[24]) is limited to three concurrent behavioral strategies (four with probe actors). We note that this limit also matches that previously proposed for human declarative working memory [22].

Despite this monitoring capacity, the binary structure of executive control in the PROBE model predicts that humans can flexibly switch back and forth between two task sets but with more difficulty across three or more task sets. Indeed, when only one task set is monitored along with the actor and with no evidence that none fit external contingencies, then the unreliability of the actor *implies* the reliability of the other task set and, consequently, its selection as an actor (Materials and Methods). In the other cases, however, especially when two or more task sets are monitored along with the actor, the unreliability of the actor does not imply the reliability of another one. In that event, a new actor is created and probed until additional evidence will possibly reveal the reliability of another task set and the rejection of the probe actor. This prediction is consistent with previous studies showing that humans are impaired in switching back and forth across three compared to two task sets, irrespective of working memory load [40]. According to the present results, this impairment reflects the binary nature rather than the monitoring capacity of human executive control.

It is worth noting that with monitoring bound *N* equal to three (or more), both the FORGET and MAX models qualitatively account for the differential performances and dependences of successive responses we observed between recurrent and open episodes. However, these differential effects result not only from increased performances in recurrent episodes but mostly from dramatic decreased performances in open episodes; both models become much more perseverative than human participants in open episodes. As shown in the Results section, both models actually reach human performances in open episodes only by monitoring a single actor task set against chance or “random behavior” (which is obtained in the FORGET model through large decay rate *ϕ*), thereby reproducing the binary control inherent to the PROBE model. In contrast to the PROBE model, however, they consequently fail to properly account for the differential performances observed between recurrent and open conditions. This provides further evidence that the binary structure of task set selection combined with the monitoring of alternative task sets are critical components of human executive function.

Accordingly, human executive function monitors up to three or four task sets and, when one appears reliable, selects it for driving behavior. Otherwise, the executive function directly creates a new task set and probes it as an actor rather than exploiting only the collection of behavioral strategies associated with current task sets. The probe actor forms a new strategy that recombines previously learned strategies stored in long-term memory and collected according to external cues (given contextual mappings). We found that recollection entropy was large (>0.7), indicating that task set creation especially prompts exploratory (random) behavior, at least when no stored strategies are specifically cued by contextual signals. In the converse case, task set creation comes to re-instantiate such externally cued strategies from long-term memory for driving behavior, even when they are not associated with current task sets. However, the PROBE model further assumes that task set creation is tested; probe actors may be discarded when, despite learning, other task sets become reliable before such probe actors. The results therefore reveal two fundamentally distinct human exploration processes: first, *uncontrolled* exploration stochastically selecting actions within actor task sets according to a softmax policy for learning behavioral strategies that maximize action utility [3],[28],[41], and second, *controlled* exploration occurring whenever no task sets appear reliable for investigating the opportunity to re-instantiate behavioral strategies stored in long-term memory or to learn new ones depending upon contextual cues.

For the sake of simplicity, the model described herein assumes that no internal alterations of action outcome utility (e.g., devaluation due to satiety) have occurred when task sets are created from behavioral strategies collected from long-term memory. Consistently, no alterations of outcome utility were induced in the present experimental protocol. To further account for possible utility alterations, selective mappings that encode action utility in behavioral strategies need to be recalibrated according to the *current* utility of action outcomes when new task sets are created. As previously proposed [42],[43], this internal recalibration is achieved through *model-based* reinforcement learning before experiencing actual action outcomes; using predictive mappings embedded in behavioral strategies for anticipating action outcomes, associated selective mappings are altered according to *current* outcome utility through standard reinforcement learning [11].

Accordingly, the PROBE model predicts that task set creation involves *model-based* reinforcement learning based on action outcome predictions, while task set execution involves *model-free* reinforcement learning based on actual action outcomes. The hypothesis is consistent with empirical findings: in extinction paradigms suppressing actual action outcomes following training, differential outcome devaluations were found to impact action selection (e.g., [42],[44]). In the PROBE model, suppressing actual action outcomes consistently triggers task set creation because the ongoing actor task set becomes unreliable. In the context of the experiment, then, task set creation comes to re-instantiate and recalibrate the learned behavioral strategy for acting (see above); its predictive mapping recalibrates the associated selective mapping according to actual outcome utility. Moreover, as adjustments to external contingencies may be faster for predictive than selective mappings (Bayesian updating versus reinforcement learning, respectively), this hypothesis may also account for contrasted devaluation effects occurring after moderate versus extensive training [45]. Thus, the PROBE model predicts that model-based reinforcement learning is involved in forming a new behavioral strategy when ongoing behavior and habit formation driven by model-free reinforcement learning become unreliable. Interestingly, the prediction differs from previous accounts assuming that the arbitration between behavioral strategies driven by model-free versus model-based reinforcement learning is based on their relative reliability [43].

We assumed that task sets represent behavior strategies comprising selective mappings encoding stimulus-response associations according to action utility, predictive mappings encoding expected action outcomes given stimuli, and contextual mappings encoding external cues predicting task set reliability. Neuroimaging studies suggest that these internal mappings are implemented in distinct frontal regions: (1) selective mappings in lateral premotor regions, because these regions are involved in learning and processing stimulus-response associations [10],[46]; (2) predictive mappings in ventromedial prefrontal regions, because these regions are engaged in learning and processing expected and actual action outcomes [47]–[50]; and (3) contextual mappings in lateral prefrontal regions, because these regions are involved in learning and selecting task sets according to contextual cues [10],[46],[51]. Neuroimaging studies further show that dorsomedial prefrontal regions evaluate the discrepancies between actual and predicted action outcomes [17],[52] and estimate the volatility of external contingencies [14]. The PROBE model thus suggests that dorsomedial prefrontal regions monitor task set reliability according to predictive mappings implemented in ventromedial prefrontal regions and volatility estimates. Lateral prefrontal regions then revise task set reliability according to contextual cues for choosing the task set driving immediate behavior (i.e., the selective mapping in the premotor cortex that specifies the responses to stimuli) [46].

The present study suggests that the prefrontal cortex monitors at most three or four task sets. The frontal network described above selects the unique task set appearing reliable for driving behavior and adjusts it according to action outcomes. When none appear reliable, this frontal network presumably enters in *controlled* exploration; a new task set is probed but initially appears unreliable, thereby requiring an additional control system to enforce or discard this probe actor. This system needs to monitor at least the second most reliable task set. When both the actor and its best alternative appear unreliable (or no alternative sets are monitored), the system enforces exploration; a new task set is created from long-term memory in the frontal network described above and drives behavior. Exploration then terminates when either this probe actor or its current best alternative becomes reliable. This putative system matches the function attributed to frontopolar regions, usually referred to as cognitive branching [53],[54]: enabling the unexpected execution of a task, while holding on and monitoring an alternative task for possible future execution. Furthermore, consistent with the notion of controlled exploration, frontopolar regions are engaged in exploratory behavior [28], long-term memory cued retrieval [55], and in the early phase of learning new behaviors [50],[56]. The PROBE model thus predicts that frontopolar regions monitor at least the reliability of the best alternative to the actor, a prediction supported by recent neuroimaging evidence [47],[57]. Finally, we found that individual variations in adaptive behavior primarily result from confirmation biases in *controlled* exploration. Consistently, the frontopolar function has been associated with individual variations in fluid intelligence [58], suggesting that fluid intelligence is associated with the ability to probe new strategies.

According to previous studies, “creativity is the epitome of cognitive flexibility. The ability to break conventional or obvious patterns of thinking, adopt new and/or higher order rules and think conceptually and abstractly is at the heart of any theory of creativity” ([59]; see also [60]). From this perspective, the PROBE model that flexibly builds task sets as abstract mental constructs referring to true or hypothetical “states of the world” for exploring and storing new behavioral rules may help us to understand creative processes underlying human adaptive behavior. In particular, the distinction mentioned above between *uncontrolled* and *controlled* exploration is similar to the distinction made in artificial intelligence between exploratory creativity (generating new low-level actions/objects) and transformational creativity (generating new higher level rules) [61],[62]. Critically, the PROBE model suggests how the human executive function regulates the exploration versus exploitation of behavioral rules and controls creativity in the service of adaptive behavior.

In summary, the results support a model of frontal lobe function integrating reasoning, learning, and creative abilities in the service of executive control and decision-making. The model suggests how the frontal lobes create and manage an expanding repertoire of flexible behavioral strategies for driving action in uncertain, changing, and open-ended environments.

## Materials and Methods

### PROBE Model

To model uncertain, variable, and open-ended environments, we assumed that in every trial *t*, there were external contingencies—that is, the possibly stochastic relationships between stimulus *s_t_*, action *a_t_*, and outcomes *o_t_* depend upon a hidden state 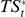 only. Hidden states are countable, potentially infinite, and vary across trials independently of stimuli and actions. Stimulus *s_t_* may be multidimensional and might include cues about current hidden states, which we refer to as contextual cues for clarity. Hidden state 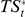 is assumed to depend only upon the preceding hidden state 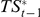 (Markov property) and contextual cues *C_t_* to depend only upon current hidden state 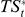.

We describe below the PROBE model computations. In Text S1, we present the statistical normative approach to the problem of task set creation based on Dirichlet Processes (see also Figure S5) and how the PROBE model approximates this statistical optimal model for the sake of biological plausibility.

#### Task sets

Task sets *TS_i_* represent possible instances of external hidden states. Each task set *i* indexes one strategy stored in long-term memory and comprises (1) a *selective* mapping 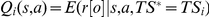 encoding expected rewarding values *r*[*o*] of outcomes *o* given action *a* and stimulus *s*; (2) a *predictive* mapping 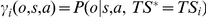 encoding the likelihood of outcome *o* given action *a* and stimulus *s*; and (3) a *contextual* mapping 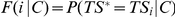 encoding the likelihood that hidden state TS* matches *TS_i_* when contextual cues C are observed (Figure S1).

#### Reliability

We assumed that the executive system monitors the reliability of at most *N* task sets. Reliability of task set *TS_i_* is the likelihood that in trial *t*, external hidden state 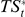 matches *TS_i_* given observations. In every trial, task set reliability is estimated in two time points: (1) before acting when stimulus *s_t_*, possibly including contextual cues *C_t_*, is observed, and (2) after action when action outcome *o_t_* is further observed. We refer to these two reliability estimates as *ex-ante* reliability *λ_i_*(*t*) and *ex-post* reliability *μ_i_*(*t*), respectively. Thus, *λ_i_*(*t*) and *μ_i_*(*t*) write as follows:
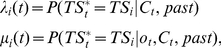
(1)where *past* refers to all other observations, including those from preceding trials. The PROBE model estimates the “absolute” reliability of task sets (i.e., the likelihood that hidden state 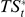 matches *TS_i_* conditionally upon observations but not upon the collection of current task sets). Such estimates require computing the likelihood that hidden state 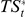 actually matches no task sets *TS_i_*. As task set reliability, this likelihood can be estimated before acting and after action. These two estimates are denoted as *λ*_0_(*t*) and *μ*_0_(*t*), respectively, and write as follows:
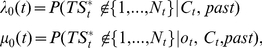
(2)where *N_t_* is the current number of task sets (*N_t_*≤*N*) and {1,…, *N_t_*} denotes the current collection of task sets.

Note that uniform predictive mapping *γ*_0_ corresponding to random predictions over action outcomes is actually an estimate of 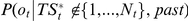. Indeed, all outcomes observed with the current collection of task sets remain equally probable, when hidden state 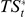 is unknown. Consequently, mapping *γ*_0_ is constant and normalized according to the number of observed outcomes: 
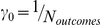
, where *N_outcomes_* counts outcomes *o* such that 
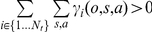
 (e.g., 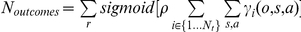 with large inverse temperature *ρ*).

For clarity, we denote 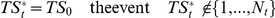. Consequently, we can write the following using Equations 1 and 2:
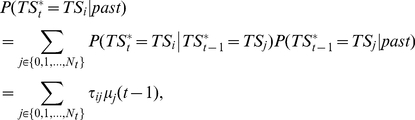
(3)where *τ_ij_* are transition probabilities from states *j* to *i*. Using standard Bayesian calculus and assuming that with no observations all task sets are presumed equally reliable (i.e., 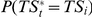 is independent of *i*), we then obtain from Equation 3 the following updating rule for ex-ante reliability:
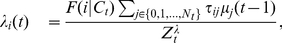
(4)where indexes 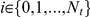 and 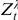 is the normalization term. Finally, we obtain the following updating rule for ex-post reliability:
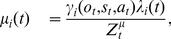
(5)where indexes 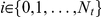 and 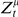 is the normalization term. Finally, transition probabilities *τ_ij_* reflect the perceived volatility *τ* of hidden states (external contingencies) across successive trials: typically 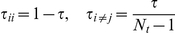, with 0<*τ*<1 and *N_t_* the current number of task sets. As previously proposed [14], volatility *τ* is estimated using a standard hidden Markov model.

#### Task set selection and creation

As described above, the PROBE model estimates the “absolute” reliability of task sets. Consequently, a minimal requirement is that the actor task set is more likely reliable than unreliable (i.e., *λ_actor_*(*t*)>1−*λ_actor_*(*t*) or equivalently, *λ_actor_*(*t*)>0.5). If a task set meets this reliability criterion, it is necessarily unique, the most reliable one, and therefore used as the actor. The criterion is necessarily fulfilled when only two task sets are monitored and *λ*_0_(*t*) is close to zero. In the other cases, the criterion is highly restrictive, so that no task sets may meet the criterion. In that case, a new task set is created to serve as an actor with prior reliability *λ_prior_*.

The new task set is created with initial selective/predictive mappings *M_new_* forming a mixture of all selective/predictive mappings stored in long-term memory and weighted according to contextual cues *C_t_*:
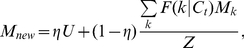
(6)where *U* denotes uniform mappings, *M_new_* and *M_k_* are selective/predictive mappings, and 
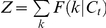
 is the normalization factor. Index *k* runs over all behavioral strategies stored in long-term memory and *η* scales *recollection entropy* (0<*η*<1), as uniform mappings *U* reflect recollection noise. Note that internal mappings with distinct index *k* may encode the same external contingencies; mixture (Equation 6) thus favors external contingencies that frequently re-occur. Given the approximations inherent to the PROBE model, more precisely, mixture (Equation 6) derives from the statistical optimal model based on Dirichlet processes (see Text S1). The mixture forms a new probe actor that is adjusted in subsequent trials through learning.

Prior reliability *λ_prior_* of the probe actor is chosen as minimizing prior information over task set reliability because no information is available to estimate it [32]. Thus, prior reliability *λ_prior_* maximizes entropy *H_t_* over reliability; that is:
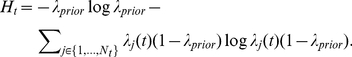
(7)Maximal entropy *H_t_* is then obtained for:
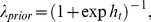
(8)where 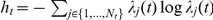 is the reliability entropy over task sets. We can verify that prior reliability *λ_prior_* ranges between 1/(*N_t_*+1) and 1/3, so that this new actor initially fails to meet the reliability criterion (i.e., *λ_prior_*≤0.5).

Consequently, the new actor is *probed* because it initially fails to meet the reliability criterion. When another task set subsequently meets the criterion while the probe actor *still* fails, the latter will be entirely *discarded*. When, conversely, learning allows the probe actor to meet the criterion while the others *still* fail, the probe phase terminates and the collection of task sets is updated as described in the main text. Note that this model favors binary compared to multiple alternative choices, because the reliability criterion is automatically fulfilled only when two task sets are monitored (and *λ*_0_(*t*)≈0; that is, the likelihood that none matches external contingencies is close to zero).

Overall, the PROBE model is an online, forward approximation of Dirichlet process mixtures [19] based on hypothesis testing on task set creation (that is, on the critical no-parametric component of Dirichlet processes; see Text S1). Hidden states 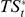 are provisionally assigned to new task sets as long as no task sets meet the reliability criterion. Conversely, hidden states are definitively assigned to task sets only when task sets meet the reliability criterion. Thus, provisional versus definitive assignments occur precisely when, in optimal statistical learning, offline backward inference is likely versus unlikely to alter previous assignments, respectively.

#### Action selection and learning

Ex-ante reliabilities *λ_i_*(*t*) serve to choose the actor. The actor selective mapping then determines the behavioral policy *P*(*a_t_*|*s_t_*) (i.e., the probability to select action *a_t_* in response to stimulus *s_t_* based on an *ε*-softmax with inverse temperature *β*):
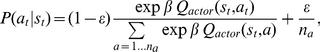
(9)where *n_a_* is the number of available actions and 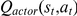 are normalized to 1 over actions (not shown in Equation 9 for clarity). After observing action outcome *o_t_*, the actor selective mapping is updated based on outcome values *r*[*o*] according to standard reinforcement learning mechanisms [11] (e.g., the simple delta rule [31]): 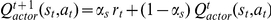, where *α_s_* is the learning rate. The actor predictive mapping simply regularizes action outcome likelihood given stimulus [13]. Contextual mappings *F*(*i*|*C_t_*) of every task set then adjust to ex-post estimates of reliability according to a standard stochastic gradient descent: 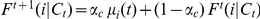, where *α_c_* is the learning rate.

#### Context-sensitivity bias

Whenever, besides regular stimuli, additional external cues change between two successive trials, participants might infer that external contingencies (i.e., hidden external states) more likely shift between these trials than others. To account for this possible bias, we considered that in every model, perceived volatility 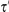 of external contingencies between such trials might be enhanced: 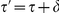, where free parameter δ≥0 is named *context-sensitivity* bias.

#### Confirmation bias

Participants might be reluctant to unselect a newly created actor set for returning to another task set. We then considered that prior reliability *λ_prior_* of such actors might be biased:
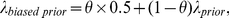
(10)where free parameter *θ* is named *confirmation* bias (0.5 is used in Equation 10 for consistency with the creation threshold).

#### Alternative models

See Text S1.

### Experimental Protocol

#### Participants

Participants were healthy, right-handed volunteers (age range, 18–35 years old) with no auditory and vision deficits and no general medical, neurological, psychiatric, or addictive history as assessed by medical examinations. Participants provided written informed consent approved by the French National Ethics Committee. Participants were paid for their participation.

#### Experimental set-up and instructions

Stimuli were visually presented arabic numbers. Participants responded to each stimulus by pressing one of four keys (Figure S2). The keys were assigned to the index and middle finger of each hand. When key presses occurred no later than 1,500 ms after stimulus onset, stimuli disappeared 100 ms after key presses and participants received audiovisual feedbacks (duration 300 ms). Feedbacks were positive or negative. A positive feedback consisted of an ascending sound and the apparition of the associated stimulus in a box representing the pressed key at the bottom of the screen. Negative feedback consisted of a descending sound only. Otherwise, stimuli were removed and no feedback was delivered. Stimulus onset asynchrony was 2,000 ms. Associations between actual stimuli, response fingers, and feedbacks were orthorgonalized and counterbalanced across participants.

Participants were instructed that feedback could be uncertain and variable and that payoffs increased with the total number of received positive feedback. No additional instructions were provided to participants.

#### Experiment 1

Experiment 1 included 22 participants (13 females). Unbeknownst to the participants, we made the following manipulations: In every trial, a “correct” response was associated with each stimulus (three possible stimuli) and led to positive feedback with a probability of 90%. All other responses led to negative feedback with a probability of 90%. Distinct stimuli were associated with distinct correct responses. Correct responses to stimuli remained unchanged over a series of successive trials, ranging from 36 to 54, named *episodes*. All correct responses to stimuli changed between two successive episodes.

The experiment included two behavioral sessions administered on 2 separate days. Each session included 25 episodes. Stimuli were pseudo-randomly chosen from the set ({1,3,5} for one session or {2,4,6} for the other session). In the *open* session, the mappings between stimuli and best responses never repeated across 24 episodes. In the last episode, the mapping from the first episode was used again, because from three stimuli and four possible responses only 24 distinct mappings can be formed (with the constraint that two distinct stimuli are associated with distinct responses). Although the *mappings* were distinct, there were considerable overlaps across the mappings. Every stimulus-response association belongs to six distinct mappings, while every pair of stimulus-response associations belongs to four distinct mappings. In order to properly define episode onsets, mappings were further organized across episodes so that there were no overlaps between two successive mappings. In the *recurrent* session, only three distinct mappings reoccurred over the episodes in a pseudo-randomized order (8/8/9 repetitions). The three mappings did not overlap (i.e., best responses to stimuli systematically differed across mappings). Transition probabilities were equalized across mappings.

Finally, episode and session order were counterbalanced across participants. Episode durations were pseudo-randomized and ranged from 36 to 54 trials, so that on average volatility of external contingencies was identical in the open and recurrent sessions (3%).

#### Experiment 2

Experiment 2 included 49 additional participants (25 females) and comprised two behavioral sessions administered on 2 consecutive days. Again, participants were not informed about the following manipulations. Stimuli were pseudo-randomly chosen from the set {1,2,3}. The first session was identical to the recurrent session described above with only one exception: stimulus colors predicted the mappings between stimuli and best responses used in each episode with 100% reliability. Two mappings were associated with unique color cues. The third one was associated with two possible color cues for assessing the effects of cue changes without episode changes (an event occurring at most once in such episodes).

The second session included 13 rehearsal episodes corresponding to the cued recurrent episodes used in the first session followed by 12 intermixed test episodes: four *control* episodes corresponding to the recurrent mapping associated with its two color cues, six *transfer* episodes corresponding to the two other recurrent mappings but now associated with new color cues, and two *open* episodes corresponding to a new mapping associated with new cues. All these mappings were fully incongruent; there were only four possible instances of such mappings, which were used in these 12 episodes. Order of episodes was counterbalanced across participants.

#### Data analyses, model fitting, and post-tests

See Text S1.

## Supporting Information

Figure S1 Architecture of task sets. The monitoring buffer comprises a limited number of task sets, each indexing a behavioral strategy stored in long-term memory and comprising a selective, predictive, and contextual mapping (M). The reliability of each task set is monitored online at two time points: right before acting (ex-ante reliability *λ_i_*) and right after perceiving action outcomes (ex-post reliability *μ_i_*); ex-ante reliability *λ_i_* is inferred from ex-post reliability in the preceding trial according to contextual cues C (given contextual models) and the perceived volatility of external contingencies (not shown); ex-post reliability *μ_i_* is inferred from ex-ante reliability preceding action according to action outcomes r (given predictive models). Ex-ante reliability serves to choose the actor driving immediate behavior. The actor selective mapping then determines the responses to stimuli. Actor selective and predictive mappings learn according to action outcomes. Contextual mappings of task sets adjust to ex-post reliability and consequently learn contextual cues C predicting task set reliability. Red indicates computations occurring within the actor set only. Arrows indicate information flows occurring within task sets. Broken arrows symbolize learning processes within internal mappings (M). Blue lines represent the associations remaining between internal mappings forming strategies stored in long-term memory and previously indexed by a task set. See Materials and Methods for notations.
(PDF)

Figure S2 Trial structure in Experiments 1 and 2. (A) First experiment. Visual stimuli were pseudo-randomly drawn from a set of three arabic numbers (e.g., (1, 3, 5)). Participants had to respond by pressing one among four possible response keys. 100 ms after participants' responses, stimuli were removed and positive or negative feedback was presented during 300 ms; positive feedback consisted of an ascending sound and stimuli appeared in a box at the bottom of the screen corresponding to the pressed key. Negative feedback consisted of descending sounds only. Stimulus onset asynchrony was 2,000 ms. (B) Second experiment. Same as Experiment 1, except that stimuli appeared in different colors. Unbeknownst to participants, stimuli colors were contextual cues associated with the different possible mappings between stimuli and best rewarding responses occurring across the experiment. Color cues changed infrequently. The figure shows the only events and external signals participants could observe in the experiments. In particular, participants had to infer any other information regarding external contingencies, including the associations between stimuli, color cues, response keys and feedback, their occurrence structure, uncertainty, and variations in the experiment.
(PDF)

Figure S3 Irrelevant contextual changes within episodes. Left, proportions of correct responses produced by context-, outcome-exploiting, and exploring participants on trials preceding and following changes in contextual cues within control episodes (Experiment 2). Contextual cues changed in Trial T, whereas the mapping between stimuli and best responses remained unchanged. Error bars are S.E.M. across participants. Right, proportions of correct responses predicted by the PROBE model for each group with parameters fitted on every participant. In every trial, predicted proportions are computed according to actual participants' responses in previous trials. Error bars are S.E.M. across participants. The model predicts that, in every group, correct responses drop off in Trial T (decreases from Trial T-1 to T, *F* = 6.7, *p*<0.001; interaction with groups, *F*<1). In every group, consistently, participants' correct responses dropped off in Trial T (decreases from Trial T-1 to T, main effect, *F*>4.9, *p*<0.001; interaction with groups, *F*<1). This result shows that in every group, participants were responsive to contextual cues as predicted by the PROBE model.
(PDF)

Figure S4 Human performances and PROBE model fit with four recurrent action sets. Shaded lines, performances from 30 healthy participants (16 females, aged 18–30 years old) in recurrent episodes plotted against the number of trials following episode onset. Shaded areas are S.E.M. across participants (detailed legend in Figure 1). The experimental session consisted of 24 recurrent episodes identical to that from Experiment 1 (see text), except that four mappings between stimuli and correct responses re-occurred pseudo-randomly across episodes. The four mappings were fully incongruent. Note that participants performed as in open episodes in Experiment 1 (see Figure 1) with no peaks of mutual dependence of successive decisions in the first trials of episodes. Lines ± error bars (mean ± S.E.M.), performances predicted by the fitted PROBE model (details in Figure 2): correct and exploratory response rates were computed in every trial according to the actual history of participants' responses. Mutual dependence of successive correct decisions predicted by the model was computed as the mutual information between two successive correct decisions produced by the model independently of actual participants' responses (one simulation for each participant). Best-fitting model parameters (mean(S.E.M.)): inverse temperature β = 35(2.3); noise ε = 0.04(.003); bound *N* = 3.4(.3); learning rate α = 0.34(.04); recollection entropy η = 0.75(.03); and confirmation bias θ = 0.34(.06). Note that the parameters are close to those from Experiment 1 (see Table S1). See Text S1 (section “Comments on Model Fits”) for additional comments regarding model and participants' behavior.
(PDF)

Figure S5 Performance of the statistical optimal model. Graphs show the best achievable performance in terms of information processing in Experiment 1. The statistical optimal model is described in Text S1, 1-Normative approach to the PROBE model, optimal statistical model. Red, recurrent episodes; green, open episodes. The best achievable performance is obtained with inferences involving at least 25 trials backwards and concentration parameter *η* = 10. Lower concentration parameters improve model performance in recurrent episodes (increased correct responses and decreased exploratory responses), but decrease model performance in open episodes. Conversely, larger concentration parameters decrease model performance in recurrent episodes but improve model performance in open episodes. Inset, human data from Figure 1 (see Figure 1 for detailed legend). In both conditions, as expected, the statistical optimal model outperforms human participants dramatically.
(PDF)

Table S1 Best fitting model parameters used in Figures 3 and 5. Mean(S.E.M.) across participants. See Materials and Methods for detailed parameter description.
(PDF)

Table S2 Best fitting parameters in the PROBE model across participants' group used in Figures 6 and 7. Mean(S.E.M.) across participants. See Materials and Methods for detailed parameter description. Boxes indicate significant differences across groups (see text).
(PDF)

Text S1 Supplementary methods.
(PDF)

## Abbreviations

RL: reinforcement learning
UM: uncertainty monitoring

## References

[1] Simon H (1997). Models of bounded rationality: empirically grounded economic reason.

[2] Kahneman D, Tversky A (2000). Choices, values and frames.

[3] Cohen J. D, McClure S. M, Yu A. J (2007). Should I stay or should I go? How the human brain manages the trade-off between exploitation and exploration.. Philos Trans R Soc Lond B Biol Sci.

[4] Glimcher P. W, Camerer C. F, Fehr E, Poldrack R. A (2009). Neuroeconomics: decision-making and the brain.

[5] Harlow H. F (1949). The formation of learning sets.. Psychological Review.

[6] Rogers R. D, Monsell S (1995). Costs of predictable switch between simple cognitive tasks.. J Exp Psychol Gen.

[7] Koechlin E, Summerfield C (2007). An information theoretical approach to prefrontal executive function.. Trends Cogn Sci.

[8] Botvinick M. M (2008). Hierarchical models of behavior and prefrontal function.. Trends Cogn Sci.

[9] Sakai K (2008). Task set and prefrontal cortex.. Annu Rev Neurosci.

[10] Badre D, Kayser A. S, D'Esposito M (2010). Frontal cortex and the discovery of abstract action rules.. Neuron.

[11] Sutton R. S, Barto A. G (1998). Reinforcement learning.

[12] O'Doherty J, Dayan P, Schultz J, Deichmann R, Friston K (2004). Dissociable roles of ventral and dorsal striatum in instrumental conditioning.. Science.

[13] Yu A, Dayan P (2005). Uncertainty, neuromodulation, and attention.. Neuron.

[14] Behrens T. E, Woolrich M. W, Walton M. E, Rushworth M. F (2007). Learning the value of information in an uncertain world.. Nat Neurosci.

[15] Doya K (2002). Metalearning and neuromodulation.. Neural Netw.

[16] Doya K, Samejima K, Katagiri K, Kawato M (2002). Multiple model-based reinforcement learning.. Neural Comput.

[17] Samejima K, Doya K (2007). Multiple representations of belief states and action values in corticobasal ganglia loops.. Ann N Y Acad Sci.

[18] Gershman S. J, Blei D. M, Niv Y (2010). Context learning, and extinction.. Psychol Rev.

[19] Doshi-Velez F (2009). The infinite partially observable markov decision process.. Adv Neural Inf Process Syst.

[20] Teh Y. W, Jordan M. I, Beal M. J, Blei D. M (2006). Hierarchical dirichlet processes.. J Am Stat Assoc.

[21] Daw N. D, Courville A (2007). The pigeon as particle filter.. Adv Neural Inf Process Syst.

[22] Cowan N, Izawa C, Ohta N (2005). Working-memory capacity limits in a theoretical context.. Human learning and memory: advances in theory and applications.

[23] Risse S, Oberauer K (2010). Selection of objects and tasks in working memory.. Quarterly J Exp Psych.

[24] Oberauer K (2010). Declarative and procedural working memory: common principles, common capacity limits?. Psychologica Belgica.

[25] Burgess N, Hitch G (2005). Computational models of working memory: putting long-term memory into context.. Trends Cogn Sci.

[26] Milner B (1963). Effects of brain lesions on card sorting.. Arch Neurol.

[27] Konishi S, Nakajima K, Uchida I, Kameyama M, Nakahara K (1998). Transient activation of inferior prefrontal cortex during cognitive set shifting.. Nat Neurosci.

[28] Daw N. D, O'Doherty J. P, Dayan P, Seymour B, Dolan R. J (2006). Cortical substrates for exploratory decisions in humans.. Nature.

[29] Dreher J-C, Berman K. F (2002). Fractionating the neural substrate of cognitive control processes.. Proc Natl Acad Sci U S A.

[30] Hyafil A, Summerfield C, Koechlin E (2009). Two mechanisms for task-switching in the prefrontal cortex.. J Neurosci.

[31] Rescorla R. A, Wagner A R, Black A. H, Prokasy W. F (1972). A theory of pavlovian conditioning: variations in the effectiveness of reinforcement and nonreinforcement.. Classical conditioning II.

[32] Jaynes E. T (1957). Information theory and statistical mechanics.. Physical Review Series II.

[33] Cowan N, Sossin W. S, Lacaille J. C, Castelluci V. F, Belleville S (2008). What are the differences between long-term, short-term, and working memory.. Progress in brain research.

[34] Ricker T. J, Cowan N, Morey C. C (2010). Working memory.. Wiley Interdisciplinary Review: Cognitive Science.

[35] Nassar M. R, Wilson R. C, Heasly B, Gold J. I (2010). An approximately Bayesian delta-rule model explains the dynamics of belief updating in a changing environment.. J Neurosci.

[36] Mathys C, Daunizeau J, Friston K. J, Stephan K. E (2011). A Bayesian foundation for individual learning under uncertainty.. Front Hum Neurosci.

[37] Braver T. S, Cole M. W, Yarkoni T (2010). Vive les differences! Individual variation in neural mechanisms of executive control.. Curr Opin Neurobiol.

[38] Mercado E (2008). Neural and cognitive plasticity: from maps to minds.. Psychological Bulletin.

[39] Gallistel C. R, Fairhurst S, Balsam P (2004). The learning curve: implications of a quantitative analysis.. Proc Natl Acad Sci U S A.

[40] Charron S, Koechlin E (2010). Divided representation of concurrent goals in the human frontal lobes.. Science.

[41] Frank M. J, Doll B. B, Oas-Terpstra J, Moreno F (2009). Prefrontal and striatal dopaminergic genes predict individual differences in exploration and exploitation.. Nat Neurosci.

[42] Balleine B. W, Dickinson A (1998). Goal-directed instrumental action: contingency and incentive learning and their cortical substrates.. Neuropharmacology.

[43] Daw N. D, Niv Y, Dayan P (2005). Uncertainty-based competition between prefrontal and dorsolateral striatal systems for behavioral control.. Nat Neurosci.

[44] Corbit L. H, Balleine B. W (2003). The role of prelimbic cortex in instrumental conditioning.. Behav Brain Res.

[45] Holland P. C (2004). Relations between Pavlovian-instrumental transfer and reinforcer devaluation.. J Exp Psychol Anim Behav Process.

[46] Koechlin E, Ody C, Kouneiher F (2003). The architecture of cognitive control in the human prefrontal cortex.. Science.

[47] Boorman E. D, Behrens T. E, Woolrich M. W, Rushworth M. F (2009). How green is the grass on the other side? Frontopolar cortex and the evidence in favor of alternative courses of action.. Neuron.

[48] Rushworth M. F. S, Behrens T. E. J (2008). Choice, uncertainty and value in prefrontal and cingulate cortex.. Nat Neurosci.

[49] O'Doherty J. P (2007). Lights, camembert, action! The role of human orbitofrontal cortex in encoding stimuli, rewards, and choices.. Ann N Y Acad Sci.

[50] Koechlin E, Danek A, Burnod Y, Grafman J (2002). Medial prefrontal and subcortical mechanisms underlying the acquisition of motor and cognitive action sequences in humans.. Neuron.

[51] Miller E. K, Cohen J. D (2001). An integrative theory of prefrontal cortex function.. Annu Rev Neurosci.

[52] Alexander W. H, Brown J. W (2010). Computational models of performance and cognitive control.. Topics in Cognitive Sciences.

[53] Koechlin E, Hyafil A (2007). Anterior prefrontal function and the limits of human decision-making.. Science.

[54] Koechlin E, Basso G, Pietrini P, Panzer S, Grafman J (1999). The role of the anterior prefrontal cortex in human cognition.. Nature.

[55] Fletcher P. C, Henson R. N (2001). Frontal lobes and human memory: insights from functional neuroimaging.. Brain.

[56] Sakai K, Hikosaka O, Miyauchi S, Takino R, Sasaki Y (1998). Transition of brain activation from frontal to parietal areas in visuomotor sequence learning.. J Neurosci.

[57] Boorman E. D, Behrens T. E, Rushworth M (2011). Counterfactual choices and learning in a neural network centered on human lateral frontopolar cortex.. PLoS Biol.

[58] Glascher J, Rudrauf D, Colom R, Paul L. K, Tranel D (2010). Distributed neural system for general intelligence revealed by lesion mapping.. Proc Natl Acad Sci U S A.

[59] Dietrich A (2004). The cognitive neuroscience of creativity.. Psychon Bull Rev.

[60] Zabelina D, Robinson M. D (2010). Creativity as flexible cognitive control.. Psychology of Aesthetics, Creativity and the Arts.

[61] Wiggins G. A (2006). A preliminary framework for description, analysis and comparison of creative system.. Knowledge-Based Systems.

[62] Boden M. A (1990). The creative mind: myths and mechanisms.

