# Supplementary material for: Reasoning, Learning, and Creativity: Frontal Lobe Function and Human Decision-Making
Source: PLoS Biol. 2012 Mar 27;10(3):e1001293. doi: 10.1371/journal.pbio.1001293 (PMC3313946; doi:10.1371/journal.pbio.1001293)
Supplement: Figure S1 — Architecture of task sets. The monitoring buffer comprises a limited number of task sets, each indexing a behavioral strategy stored in long-term memory and comprising a selective, predictive, and contextual mapping (M). The reliability of each task set is monitored online at two time points: right before acting (ex-ante reliability λi) and right after perceiving action outcomes (ex-post reliability μi); ex-ante reliability λi is inferred from ex-post reliability in the preceding trial according to contextual cues C (given contextual models) and the perceived volatility of external contingencies (not shown); ex-post reliability μi is inferred from ex-ante reliability preceding action according to action outcomes r (given predictive models). Ex-ante reliability serves to choose the actor driving immediate behavior. The actor selective mapping then determines the responses to stimuli. Actor selective and predictive mappings learn according to action outcomes. Contextual mappings of task sets adjust to ex-post reliability and consequently learn contextual cues C predicting task set reliability. Red indicates computations occurring within the actor set only. Arrows indicate information flows occurring within task sets. Broken arrows symbolize learning processes within internal mappings (M). Blue lines represent the associations remaining between internal mappings forming strategies stored in long-term memory and previously indexed by a task set. See Materials and Methods for notations. (PDF) [file pbio.1001293.s001.pdf]

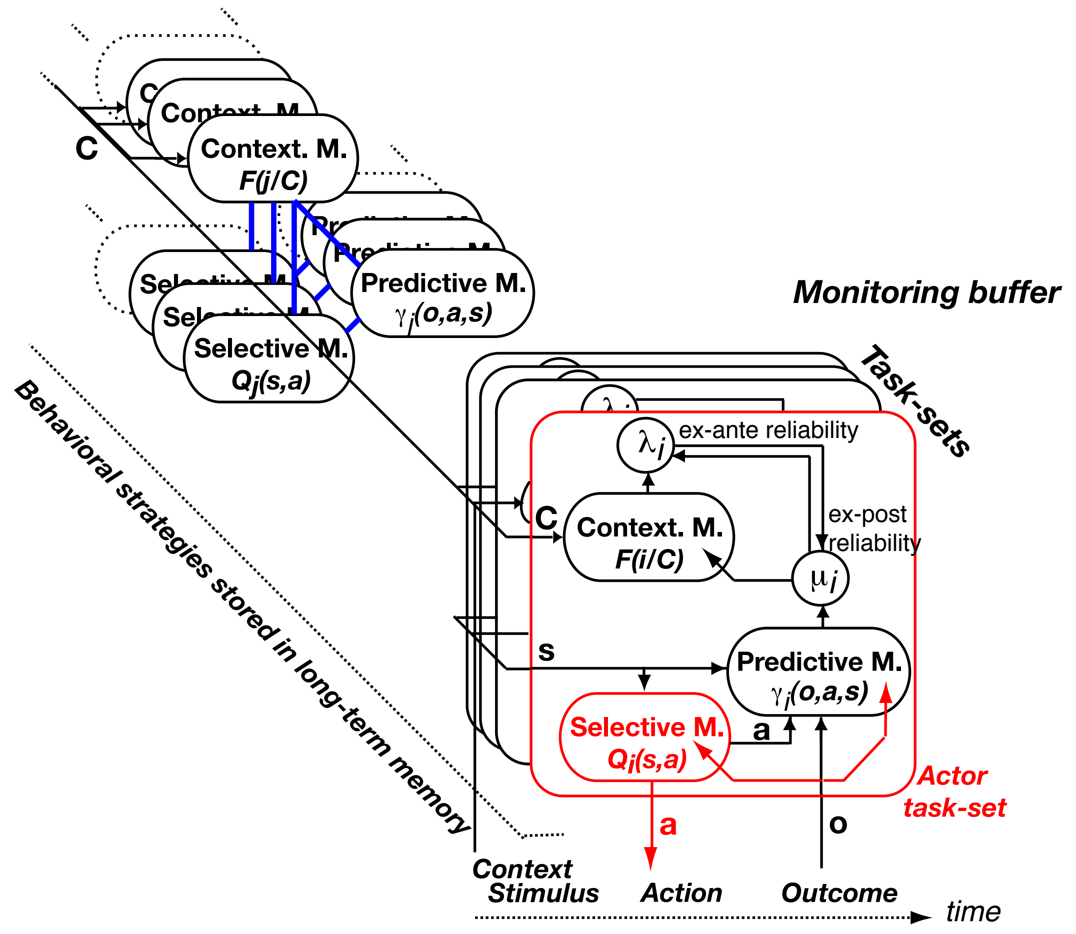

Fig. S1. **Architecture of task sets**. The monitoring buffer comprises a limited number of task sets, each indexing a behavioral strategy stored in long-term memory and comprising: a selective, predictive and contextual mapping (M.). The reliability of each task set is monitored online at two time points: right before acting (ex-ante reliability λ_i) and right after perceiving action outcomes (ex-post reliability μ_i): ex-ante reliability λ_i is inferred from ex-post reliability in the preceding trial according to contextual cues C (given contextual models) and the perceived volatility of external contingencies (not shown); ex-post reliability μ_i is inferred from ex-ante reliability preceding action according to action outcomes r (given predictive models). Ex-ante reliability serves to choose the actor driving immediate behavior. The actor selective mapping then determines the responses to stimuli. Actor selective and predictive mappings learns according to action outcomes. Contextual mappings of task sets adjust to ex-post reliability and consequently learns contextual cues C predicting task set reliability. Red indicates computations occurring within the actor set only. Arrows indicate information flows occuring within task sets. Broken arrows symbolize learning processes within internal mappings (M.). Blue lines represent the associations remaining between internal mappings forming strategies stored in long-term memory and previously indexed by a task set. See Methods for notations.
