# Supplementary material for: Reasoning, Learning, and Creativity: Frontal Lobe Function and Human Decision-Making
Source: PLoS Biol. 2012 Mar 27;10(3):e1001293. doi: 10.1371/journal.pbio.1001293 (PMC3313946; doi:10.1371/journal.pbio.1001293)
Supplement: Figure S2 — Trial structure in Experiments 1 and 2. (A) First experiment. Visual stimuli were pseudo-randomly drawn from a set of three arabic numbers (e.g., (1, 3, 5)). Participants had to respond by pressing one among four possible response keys. 100 ms after participants' responses, stimuli were removed and positive or negative feedback was presented during 300 ms; positive feedback consisted of an ascending sound and stimuli appeared in a box at the bottom of the screen corresponding to the pressed key. Negative feedback consisted of descending sounds only. Stimulus onset asynchrony was 2,000 ms. (B) Second experiment. Same as Experiment 1, except that stimuli appeared in different colors. Unbeknownst to participants, stimuli colors were contextual cues associated with the different possible mappings between stimuli and best rewarding responses occurring across the experiment. Color cues changed infrequently. The figure shows the only events and external signals participants could observe in the experiments. In particular, participants had to infer any other information regarding external contingencies, including the associations between stimuli, color cues, response keys and feedback, their occurrence structure, uncertainty, and variations in the experiment. (PDF) [file pbio.1001293.s002.pdf]

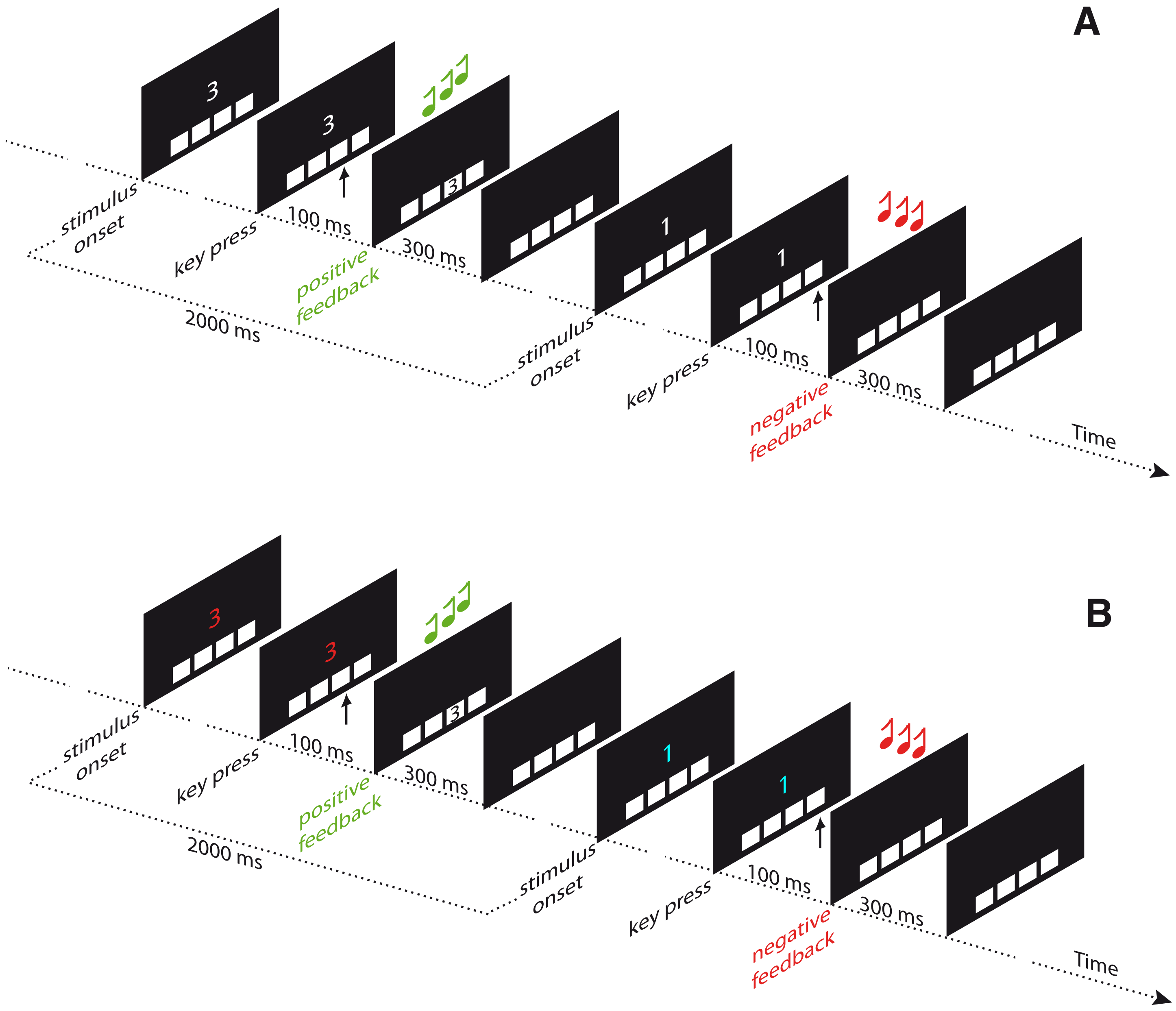

Fig. S2. Trial structure in experiment 1 and 2. **A**, first experiment. Visual stimuli were pseudorandomly drawn from a set of three arabic numbers, *e.g.* (1,3, 5). Subjects had to respond by pressing one among four possible response keys. 100 ms after subjects' responses, stimuli were removed and a positive or negative feedback was presented during 300 ms: positive feedbacks consisted of an ascending sound and stimuli appeared in a box at the bottom of the screen corresponding to the pressed key. Negative feedbacks consisted of descending sounds only. Stimulus onset asynchrony was 2000 ms. **B**, second experiment. Same as experiment 1, except that stimuli appeared in different colors. Unbeknownst to subjects, stimuli colors were contextual cues associated with the different possible mappings between stimuli and best rewarding responses occurring across the experiment. Color cues changed infrequently. The figure shows the only events and external signals subjects could observe in the experiments.
In particular, subjects had to infer any other information regarding external contingencies, including the associations between stimuli, color cues, response keys and feedbacks, their occurence structure, uncertainty and variations in the experiment.
