# Supplementary material for: Reasoning, Learning, and Creativity: Frontal Lobe Function and Human Decision-Making
Source: PLoS Biol. 2012 Mar 27;10(3):e1001293. doi: 10.1371/journal.pbio.1001293 (PMC3313946; doi:10.1371/journal.pbio.1001293)
Supplement: Figure S3 — Irrelevant contextual changes within episodes. Left, proportions of correct responses produced by context-, outcome-exploiting, and exploring participants on trials preceding and following changes in contextual cues within control episodes (Experiment 2). Contextual cues changed in Trial T, whereas the mapping between stimuli and best responses remained unchanged. Error bars are S.E.M. across participants. Right, proportions of correct responses predicted by the PROBE model for each group with parameters fitted on every participant. In every trial, predicted proportions are computed according to actual participants' responses in previous trials. Error bars are S.E.M. across participants. The model predicts that, in every group, correct responses drop off in Trial T (decreases from Trial T-1 to T, F = 6.7, p<0.001; interaction with groups, F<1). In every group, consistently, participants' correct responses dropped off in Trial T (decreases from Trial T-1 to T, main effect, F>4.9, p<0.001; interaction with groups, F<1). This result shows that in every group, participants were responsive to contextual cues as predicted by the PROBE model. (PDF) [file pbio.1001293.s003.pdf]

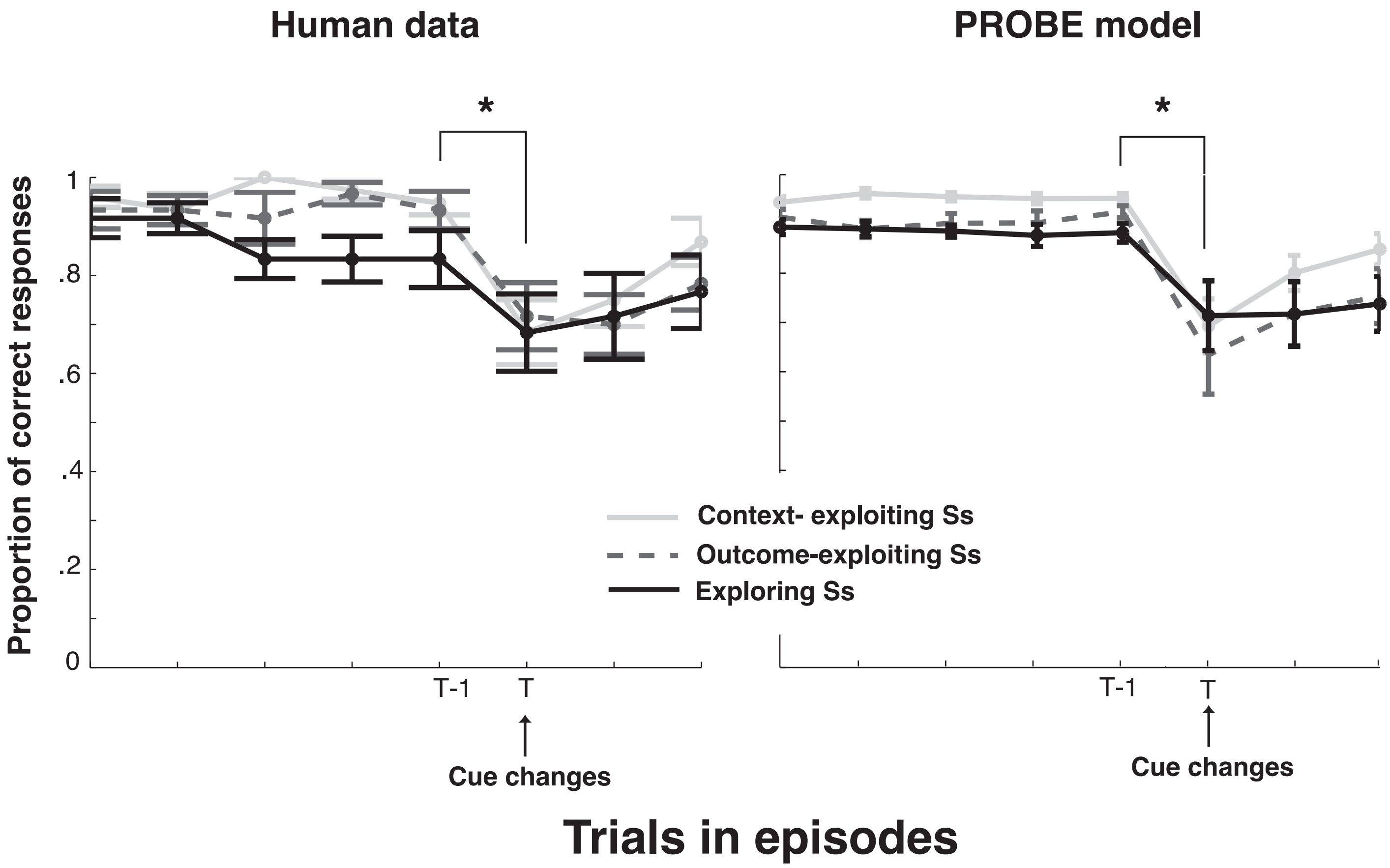

Fig. S3. Irrelevant contextual cue changes within episodes.
Left, proportions of correct responses produced by context-, outcome- exploiting and exploring subjects on trials preceding and following changes in contextual cues within control episodes (experiment 2): Contextual cues changed in trial T, whereas the mapping between stimuli and best responses remained unchanged. Error bars are SEM across subjects. **Right**, proportions of correct responses predicted by the PROBE model for each group with parameters fitted on every subject. In every trial, predicted proportions are computed according to actual subjects' responses in previous trials. Error bars are SEM across subjects. The model predicts that in every group, correct responses drop off in trial T (decreases from trial T-1 to T: $F=6.7$, $P<0.001$; interaction with groups: $F<1$). In every group, consistently, subjects' correct responses dropped off in trial T (decreases from trial T-1 to T: main effect $F>4.9$, $P<0.001$; interaction with groups $F<1$). This result shows that in every group, subjects were responsive to contextual cues as predicted by the PROBE model.
