# Supplementary material for: Reasoning, Learning, and Creativity: Frontal Lobe Function and Human Decision-Making
Source: PLoS Biol. 2012 Mar 27;10(3):e1001293. doi: 10.1371/journal.pbio.1001293 (PMC3313946; doi:10.1371/journal.pbio.1001293)
Supplement: Figure S4 — Human performances and PROBE model fit with four recurrent action sets. Shaded lines, performances from 30 healthy participants (16 females, aged 18–30 years old) in recurrent episodes plotted against the number of trials following episode onset. Shaded areas are S.E.M. across participants (detailed legend in Figure 1). The experimental session consisted of 24 recurrent episodes identical to that from Experiment 1 (see text), except that four mappings between stimuli and correct responses re-occurred pseudo-randomly across episodes. The four mappings were fully incongruent. Note that participants performed as in open episodes in Experiment 1 (see Figure 1) with no peaks of mutual dependence of successive decisions in the first trials of episodes. Lines ± error bars (mean ± S.E.M.), performances predicted by the fitted PROBE model (details in Figure 2): correct and exploratory response rates were computed in every trial according to the actual history of participants' responses. Mutual dependence of successive correct decisions predicted by the model was computed as the mutual information between two successive correct decisions produced by the model independently of actual participants' responses (one simulation for each participant). Best-fitting model parameters (mean(S.E.M.)): inverse temperature β = 35(2.3); noise ε = 0.04(.003); bound N = 3.4(.3); learning rate α = 0.34(.04); recollection entropy η = 0.75(.03); and confirmation bias θ = 0.34(.06). Note that the parameters are close to those from Experiment 1 (see Table S1). See Text S1 (section “Comments on Model Fits”) for additional comments regarding model and participants' behavior. (PDF) [file pbio.1001293.s004.pdf]

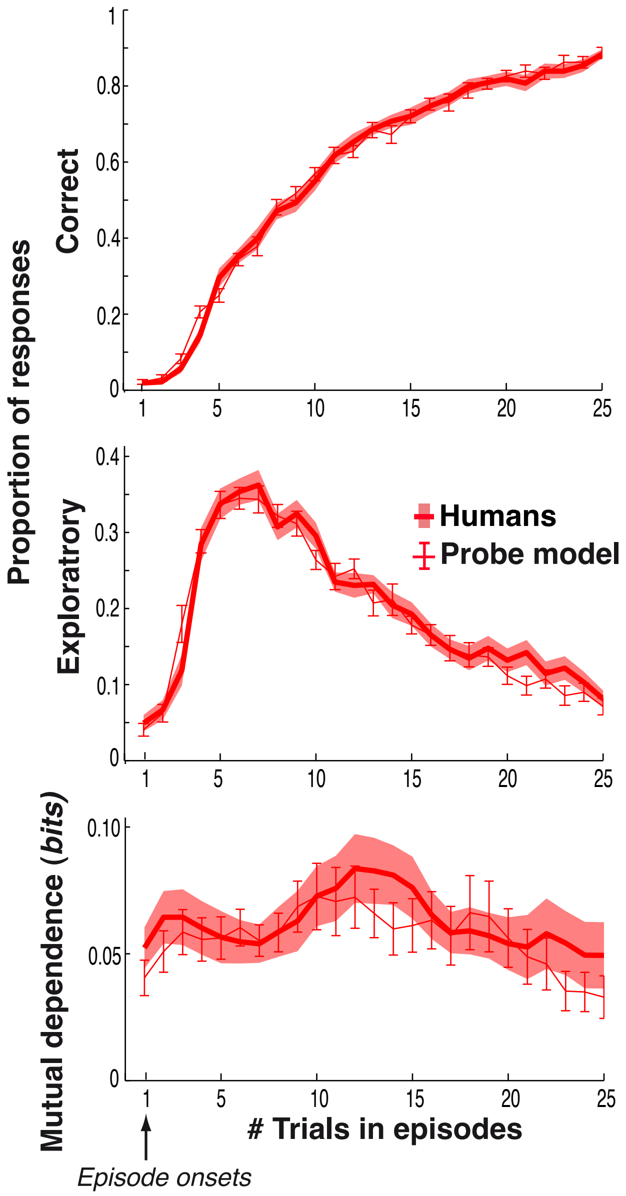

Fig. S4. Human performances and PROBE model fit with 4 recurrent action sets. Shaded lines, performances from 30 healthy subjects (16 females, aged 18-30 y/o) in recurrent episodes plotted against the number of trials following episodes onsets. Shaded areas are s.e.m. across subjects (detailed legend in **Fig. 1**). The experimental session consisted of 24 recurrent episodes identical to that from Exp. 1 (see text), except that four mappings between stimuli and correct responses re-occured pseudo-randomly across episodes. The four mappings were fully incongruent. Note that subjects performed as in open episodes in Exp. 1 (see **Fig. 1**) with no peaks of mutual dependence of successive decisions in the first trials of episodes. **Lines +/- error bars** (mean +/- s.e.m.), performances predicted by the fitted PROBE model (details in **Fig. 2**): correct and exploratory response rates were computed in every trial according to the actual history of subjects's responses. Mutual dependence of successive correct decisions predicted by the model was computed as the mutual information between two successive correct decisions produced by the model independently of actual subjects' responses (1 simulation for each subject). Best-fitting model parameters (mean(s.e.m.)): inverse temperature β=35(2.3); noise ε=0.04(.003); bound N=3.4(.3); learning rate α=0.34(.04); recollection entropy η=0.75(.03); confirmation bias θ=0.34(.06). Note that the parameters are close to those from Exp. 1 (see **Table S1**) See **Supporting Information** (section Comments on model fits) for additional comments regarding model and subjects' behavior.
