# Supplementary material for: Reasoning, Learning, and Creativity: Frontal Lobe Function and Human Decision-Making
Source: PLoS Biol. 2012 Mar 27;10(3):e1001293. doi: 10.1371/journal.pbio.1001293 (PMC3313946; doi:10.1371/journal.pbio.1001293)
Supplement: Figure S5 — Performance of the statistical optimal model. Graphs show the best achievable performance in terms of information processing in Experiment 1. The statistical optimal model is described in Text S1, 1-Normative approach to the PROBE model, optimal statistical model. Red, recurrent episodes; green, open episodes. The best achievable performance is obtained with inferences involving at least 25 trials backwards and concentration parameter η = 10. Lower concentration parameters improve model performance in recurrent episodes (increased correct responses and decreased exploratory responses), but decrease model performance in open episodes. Conversely, larger concentration parameters decrease model performance in recurrent episodes but improve model performance in open episodes. Inset, human data from Figure 1 (see Figure 1 for detailed legend). In both conditions, as expected, the statistical optimal model outperforms human participants dramatically. (PDF) [file pbio.1001293.s005.pdf]

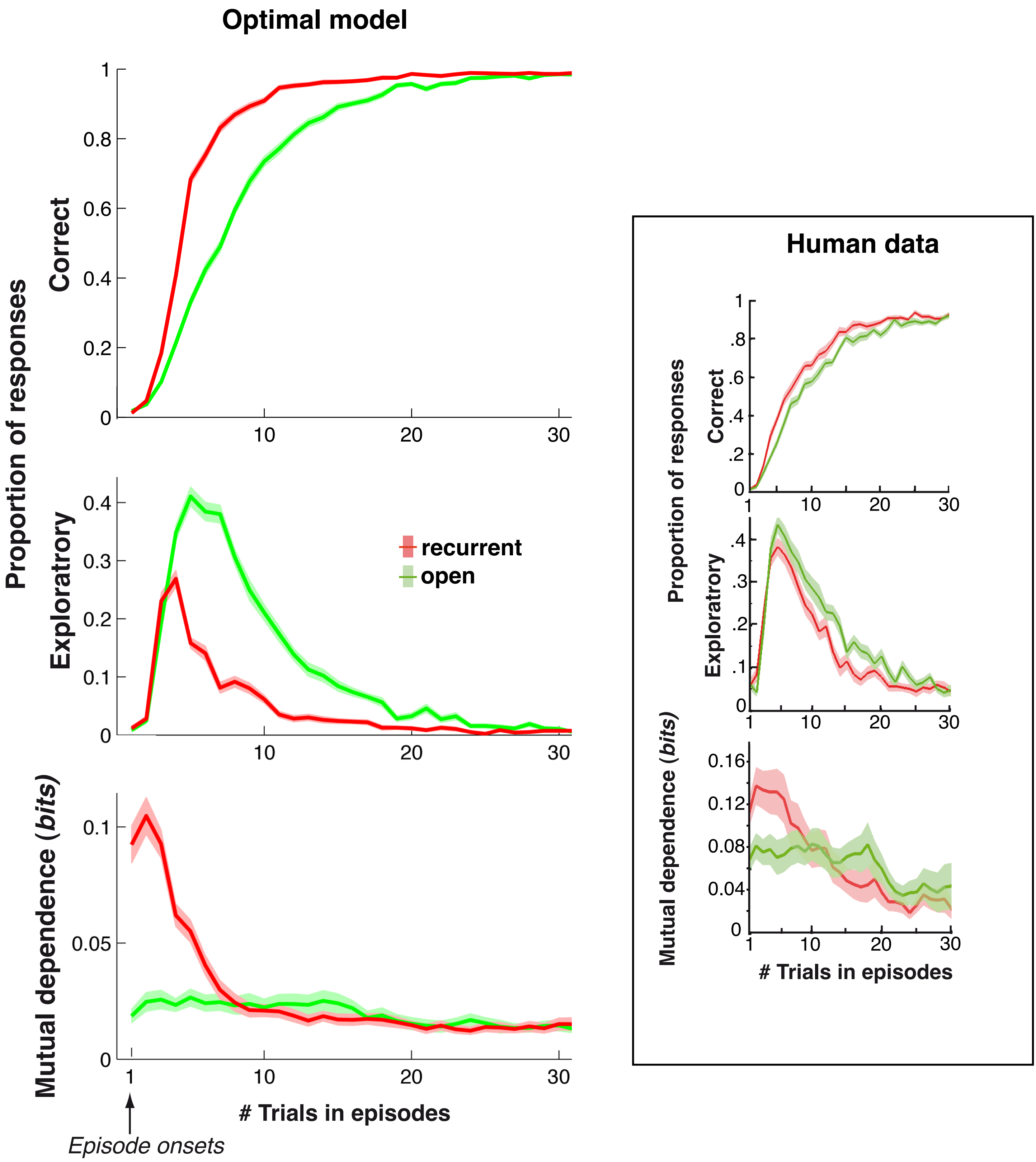

Fig. S5. Performance of the statistical optimal model in Exp. 1. Graphs show the best achievable performance in terms of information processing in Exp. 1. The statistical optimal model is described in Supplementary Methods, 1-Normative approach to the PROBE model, optimal statistical model. **Red**, recurrent episodes. **Green**, open episodes. The best achievable performance is obtained with inferences involving at least 25 trials backwards and concencatrion parameter η=10. Lower concentration parameters improve model performance in recurrent episodes (increased correct responses and decreased exploratory responses), but decrease model performance in open episodes. Larger concentration parameters, conversely, decrease model performance in recurrent episodes but improves model performance in open episodes **Inset**: human data from **Fig. 1** (see **Fig. 1** for detailed legend). In both conditions, as expected, the statistical optimal model outperforms human subjects dramatically.
