# Supplementary material for: Reasoning, Learning, and Creativity: Frontal Lobe Function and Human Decision-Making
Source: PLoS Biol. 2012 Mar 27;10(3):e1001293. doi: 10.1371/journal.pbio.1001293 (PMC3313946; doi:10.1371/journal.pbio.1001293)
Supplement: Table S1 — Best fitting model parameters used in Figures 3 and 5. Mean(S.E.M.) across participants. See Materials and Methods for detailed parameter description. (PDF) [file pbio.1001293.s006.pdf]

Table S1. Best-fitting model parameters used in Figs. 3 & 5.
Mean(SEM) across subjects. See **Materials and Methods** for detailed parameter description.

Models	inv. temp. β	noise ε	bound N	learning rate α_s	learning rate α_c	Recoll. entropy η	decay rate φ	inv. temp. β'	mixture rate ω	context-sensit. bias δ	confirm. bias θ
Exp. 1											
RL	23(3)	.00	-	.68(.02)	n/a	-	-	n/a	n/a	-	-
FORGET	47(2)	.00	2.2(.16)	.48(.02)	n/a	-	.14(.01)	21(3)	-	n/a	-
MAX	31(3)	.02(.01)	1.4(.14)	.50(.03)	n/a	.60(.09)	-	-	-	n/a	.55(.07)
PROBE	32(2)	.01(.003)	3.3(.3)	.41(.03)	n/a	.72(.07)	-	-	-	n/a	.74(.12)
Exp. 2											
RL	18(2)	.07(.01)	-	.74(.06)	.24(.02)	-	-	28(3)	.79(.03)	-	-
FORGET	12(.4)	.04(.01)	2.0(.02)	.81(.02)	.03(.002)	-	.01(.002)	26(2)	-	.43(.04)	-
MAX	15(2)	.12(.01)	4.6(.2)	.34(.03)	.01(.002)	.78(.04)	-	-	-	.55(.04)	.03(.01)
PROBE	25(2)	.05(.01)	3.2(.3)	.18(.01)	.006(.002)	.84(.02)	-	-	-	.55(.04)	.71(.06)

n/a: parameters cannot be estimated.
-: no parameter in the model.
