# Supplementary material for: Reasoning, Learning, and Creativity: Frontal Lobe Function and Human Decision-Making
Source: PLoS Biol. 2012 Mar 27;10(3):e1001293. doi: 10.1371/journal.pbio.1001293 (PMC3313946; doi:10.1371/journal.pbio.1001293)
Supplement: Table S2 — Best fitting parameters in the PROBE model across participants' group used in Figures 6 and 7. Mean(S.E.M.) across participants. See Materials and Methods for detailed parameter description. Boxes indicate significant differences across groups (see text). (PDF) [file pbio.1001293.s007.pdf]

Table S2. Best-fitting parameters in the PROBE model across subjects' groups used in Figs. 6 & 7. Mean(SEM) across subjects. See **Materials and Methods** for detailed parameter description. Boxes indicate significant differences across groups (see text).

Subjects' groups	inv. temp. β	noise ε	bound N	learning rate α_s	learning rate α_c	Recoll. entropy η	context-sensit. Bias δ	confirmation bias θ
**Exploiting vs. exploring Ss***								
Exploiting Ss	25(2)	.04(.01)	3.3(.3)	.24(.02)	n/a	.82(.02)	n/a	.57(.06)
Exploring Ss	29(2)	.05(.01)	3.0(.3)	.27(.04)	n/a	.77(.06)	n/a	1.03(.08)
**Cont.- vs. Out.- exploit. Ss****								
Context-exploit. Ss	21(2)	.03(.004)	3.5(.5)	.19(.03)	.01(.003)	.77(.03)	.71(.04)	.61(.09)
Outcome-exploit. Ss	21(3)	.06(.01)	3.5(.4)	.18(.03)	.004(.002)	.86(.02)	.47(.05)	.55(.08)

*: subjects from both experiments.

**: subjects from experiment 2.

n/a: contextual parameters α_c and δ cannot be estimated in Experiment 1.
