# Supplementary material for: Reasoning, Learning, and Creativity: Frontal Lobe Function and Human Decision-Making
Source: PLoS Biol. 2012 Mar 27;10(3):e1001293. doi: 10.1371/journal.pbio.1001293 (PMC3313946; doi:10.1371/journal.pbio.1001293)
Supplement: Text S1 — Supplementary methods. (PDF) [file pbio.1001293.s008.pdf]

SUPPLEMENTARY METHODS

1- Normative approach to the PROBE model

In this section, we briefly present the optimal statistical approach to the problem of task set creation. We then explain how the PROBE model approximates this optimal statistical model.

Optimal statistical model

The optimal statistical model is based on non-parametric Bayesian inferences creating sets on demands and known as Dirichlet Processes [1]. The model involves the following priors: (1) the model explicitly tracks hidden states switches between two consecutives trials with a binary indicator following a Bernouilli distribution with external volatility τ as parameter, where external volatility τ follow a Beta distribution to be inferred; (2) when a switch is inferred between trial t and t+1, the model assumes that given hidden state in trial t, the hidden state in trial t+1 follow a Dirichlet process with concentration parameter η: hidden states are assumed to re-occur in proportion of previous re-occurrences, whereas a new hidden state is instantiated in proportion of η. The model then infers ex-ante reliability $\Lambda(t+1)$ over hidden states, *i.e.* the distribution of task sets in trial t+1 given past action outcomes and priors about task set structures (see below). The model then chooses the most likely correct action in response to stimulus s_{t+1} by marginalizing over task sets. As shown by computer simulations, the model behavior remains virtually unchanged when alternatively, action selection involves two stages: first choosing the most reliable task set then the correct action according to this task set.

We implemented this model with priors about task set structures matching the generative model of the experimental task: each stimulus was associated with only one correct response and positive/negative feedbacks were noisy with white noise independent of hidden states (i.e. trials), stimuli, actions and outcomes. Accordingly, the model implementation assumed that positive feedbacks for correct and incorrect actions are given with probability p and 1-p, respectively, where p follows a Beta distribution inferred from action outcomes. These priors were chosen so that the normative model behavior provides the best achievable performance in the experimental task in terms of information processing. The priors also correspond to the most parsimonious interpretation of instructions given to subjects. With these priors, the number of possible task sets becomes finite and equal to 24. We therefore implemented the finite version of Dirichlet processes that reduces to the Dirichlet distribution with concentration parameter η: whenever a switch of hidden states is inferred, the larger η the more the model selects task sets randomly among the 24 possible ones regardless of their previous re-occurrences.

In this normative model, the history of task set assignments and creation is systematically revised using multiple backward-forward runs whenever in every trial T, a new action outcome is observed, so that the model statistical inferences match the memorized history of observed external contingencies. We computed the model statistical inferences using Gibbs sampling [2]. Gibbs sampling is an *offline* algorithm that according to every new action outcome in trial T, re-samples volatility τ, reward probability p and in a backward fashion, the history of inferred hidden states and switches in previous trials $t<T$.

Computer simulations show that in the first experiment, the optimal model performance (=86% of correct responses over both sessions) is virtually reached when forward-backward

inferences involve at least ~25 trials backwards (note that the optimal PROBE model performance is 80%). As expected, the optimal model outperforms human subjects in both the recurrent and open condition (see **Fig. S5**).

Computer simulations further show that for reaching human average performances in the first experiment (M+/-SE=77% +/- 0.6%), the optimal statistical model needs to involve at least ~6 trials backwards in forward-backward inferences. In the open session, consequently, the model needs to track and update backwards up to ~150 pieces of information in every trial (~24 created task-sets x 6 backward trials) for reaching human performances. The optimal statistical model thus requires memory capacities that clearly overcome human working-memory capacities. Accordingly, forward-backward inferences in Dirichlet processes that involve an increasing number of task sets have little biological plausibility.

PROBE model approximation

For the sake of biological plausibility, the PROBE model approximates the statistical optimal model in two ways: backward inferences are restricted to task-set creation (the critical non-parametric component of Dirichlet processes) and forward inferences involve only a limited number of concurrent task sets. For that purpose, the PROBE model is based on *estimating* the likelihood that no monitored task sets match the current hidden state in order to infer *online* the opportunity to create new task sets. Estimating this likelihood (*i.e.* $\mu_0(t)$, $\lambda_0(t)$, see **Materials and Methods**) then yields to the notion of "absolute" reliability of task sets: namely the estimate of likelihood $\lambda_i(T)$ that task set i match current hidden state i^* conditionally upon past observations and critically, not upon the collection of current task-sets).

In every trial T, consequently, a task set i may appear to be reliable, *i.e.* more likely reliable than unreliable, ($\lambda_i(T) > 1-\lambda_i(T)$, *i.e.* $\lambda_i(T)>0.5$), in which case the other task sets appear to be unreliable (because reliability signals sum up to 1 over task sets). When such a reliable task set exists, it is therefore assigned to trial T for driving behavior. When in trial T, conversely, no reliable task set exists, the behavior in trial T is initially driven by the mixture of learned behavioral strategies stored in long-term memory that is derived from the optimal statistical model (see below). Accordingly, this mixture forms a new, probe actor that is adjusted in subsequent trials through learning.

Creating new task-sets then logically increases the number of task sets that are concurrently monitored. Biological plausibility further requires that this number remains bound (with *bound N*) and consequently requires "deciding" which task sets to monitor. The PROBE model simply assumes that only the *last N-1 assigned* task sets (*i.e.* the last *N-1* reliable task sets) are monitored along with the actor. As a result, a new probe actor may be entirely discarded when it still remains unreliable at the time another task set becomes reliable and is assigned to drive behavior.

The resulting process corresponds to the notion of *hypothesis testing* on task set creation, *i.e.* a basic form of backward inference that possibly runs over large series of trials. The process ensures that only behavioral strategies that have been reliable and assigned to trials are stored in long-term memory. This property ensures that the mixture of behavioral strategies shaping new probe actors properly approximates the optimal statistical learning model as explained below. In sum, task set assignments to trials are based on forward inferences. However, such

assignments are definitive only when they appear to be reliable, so that in the statistical optimal model, they would be unlikely to be revised in backward inferences.

Task set creation and mixture of behavioral strategies. Probe task sets are created with initial selective mapping $M_{new}(S)$ (and predictive mapping $M_{new}(P)$) formed from the mixture of selective mappings $M_k(S)$ (predictive mappings $M_k(P)$, respectively) stored in long-term memory and weighted according to contextual cues C_t:

$$M_{new}(.) = \eta U + (1-\eta)\frac{\sum_k F(k|C_t)M_k(.)}{Z}, \qquad \text{(Eq. S1)}$$

where U denote uniform mappings and $Z = \sum_k F\left(k|C_t\right)$ the normalization factor. Indexes k runs over all behavioral strategies stored in long-term memory. Parameter η scales *recollection entropy* $(0 \le \eta \le 1)$. The mixture in (Eq. S1) derives from the statistical optimal model based on Dirichlet processes described above. To clarify this point, consider for example the formation of new predictive mapping M_{new}(P) = $\gamma_{new}(o,s,a)$. When this mapping is created, it writes as follow:

$$\gamma_{new}(o,s,a) = P(o/s,a,X,C)$$

where X is event "task set creation" with contextual cues C. Bayesian derivations then show that:

$$\gamma_{new}(o,s,a) = P(o/s,a,X,i^* = i_0)P(i^* = i_0/X) + [1 - P(i^* = i_0/X)]\sum_i P(o/s,a,X,i^* = i)\omega(i,C)$$

(Eq.S2)

with $\omega(i,C) = \dfrac{P(X/i^* = i)F(i/C)}{\sum_j P(X/i^* = j)F(j/C)}$. In Eq. S2, i,j denote hidden states that previously occurred and therefore correspond to behavioral strategies stored in long-term memory; $F(i/C)$ denotes contextual models; i^* denotes the actual hidden state when event X occurs; and $i^*=i_0$ denotes an hidden state that never occurred (no instances in long-term memory). Eq. S2 is obtained from the model assumption that external contingencies and contextual cues depend only upon actual hidden state i^* (see **Materials and Methods**). Note that:

- $P(i^*=i_0/X)$ is the probability that an entirely new hidden state occurs, when a task set is created. This probability is a parameter named *recollection entropy* η that corresponds to the *concentration parameter* in Dirichlet processes (see above). $P(o/.,X, i^*=i_0)$ is the associated predictive mapping and is therefore uniform U.

- $P(o/s,a,X, i^*=i) = P(o/s,a, i^*=i)=\gamma_i(o,s,a)$ is the predictive mapping associated with hidden state i (X is removed because external contingencies depend only upon actual hidden states).

- $P(X/i^*=i)$ is the probability of task set creation, when actual hidden state is i. Because task set creation consists of creating a new instance of behavioral strategy in long-term memory, this probability is precisely the proportion of behavioral strategies stored in long-term memory and associated with hidden state i. Consequently, we have:

$$\sum_i P(o/s,a,X,i^*=i)\omega(i,C) = \frac{\sum_k \gamma_k(o,s,a)F(k/C)}{\sum_k F(k/C)}$$

where k runs over *all behavioral strategies* stored in long-term memory. We then get from (Eq. S2) the proposed mixture:

$$\gamma_{new}(o,s,a) = U\eta + (1-\eta)\frac{\sum_k \gamma_k(o,s,a)F(k/C)}{\sum_k F(k/C)}. \qquad \text{(Eq. S3)}$$

The same calculus holds for selective mappings. In the present protocol and given the approximations inherent to the PROBE model, the proposed mixture is thus the optimal *shaping* of new actors from long-term memory, knowing that long-term memory is not an exhaustive representation of external states (open environments).

Note finally that the aim of the paper is not to provide a comprehensive description of long-term memory retrieval, which in general might include additional temporal factors such as the recency and duration of occurrences of external contingencies: as mentioned above, these factors are irrelevant in the present experimental paradigm and were ignored to avoid unnecessary sophistication.

2-Alternative models

MAX model. The MAX model is identical to the PROBE model, except that the assumption of hypothesis testing on task set creation is removed. Thus, the most reliable task set is the actor, provided that it remains more reliable than random behavior, *i.e.* $\lambda_{actor}(t) > \lambda_0(t)$ or equivalently, more likely that no task sets match current hidden state. In the converse case, a new task set is created to serve as actor with now prior reliability λ_{prior} equal to random prediction reliability $\lambda_0(t)$. This new task set directly updates task set collection, because its prior reliability is the largest one. There is therefore no hypothesis testing. Otherwise, the MAX model is identical to the PROBE model. The MAX model is equivalent to the basic online, forward approximation (one-particule filtering) of Dirichlet process mixtures[3], when bound N is infinite and recollection entropy η is 1.

Confirmation bias θ was defined as follow: $\lambda_{biased\ prior} = \theta \times 1 + (1-\theta)\lambda_{prior}$, because value 0.5 has no more meaning in this model.

FORGET model. The FORGET model is similar to the MAX model except that the assumption of task set creation is removed. Thus, the collection of task sets is fixed and corresponds to monitoring capacity N. As external states are potentially infinite, task set

reliability therefore represents relative evidence across distinct behavioral strategies rather than external states. Accordingly, only relative estimates of task set reliability are meaningful:

$$\lambda_i(t) = \frac{1}{Z_\lambda^t} F\left(i \middle| C_t\right) \sum_{j=1\ldots N} \tau_{ij} \mu_j(t-1), \quad i \in \left\{1,\ldots,N\right\}$$

$$\mu_i(t) = \frac{\gamma_i(o_t, s_t, a_t)\lambda_i(t)}{Z_\mu^t}, \quad i \in \left\{1,\ldots,N\right\}$$

where $\left\{1,\ldots,N\right\}$ denotes task set collection (fixed). This model assumes that this fixed, possibly very large collection coding for alternative behavioral strategies is used for driving behavior. Accordingly, selective and predictive mappings M associated with task sets are assumed to decay with time into uniform mappings whenever they are not chosen as actors: $M^{t+1} = \varphi U + (1-\varphi)M^t$, where U denotes uniform mappings and φ the decay rate $(0 \le \varphi < 1)$. Because reliability estimates are relative to each other, the actor is then chosen according to a softmax on ex-ante reliability $\lambda_i(t)$ with inverse temperature β'. Otherwise, the FORGET model has the same free parameters as the PROBE and MAX model, except that decay rate φ and inverse temperature β' replace recollection entropy η and confirmation bias θ, respectively.

Because in this model task set reliability therefore represents relative evidence across distinct behavioral strategies rather than external states, we properly assumed that in contrast to the MAX and PROBE model, learning/updating of selective/predictive mappings operates in all task sets i in proportion to ex-post reliability $\mu_i(t)$ [note that actually, this assumption had no influences on model fits, because in the present study, the best fitting FORGET model involved large decay rate φ (see Results)].

Pure reinforcement learning (RL) model. The FORGET, MAX and PROBE models were compared to a pure reinforcement learning model including no reliability monitoring. This RL model comprises only selective mapping $Q(s,a)$ representing stimulus-response associations and in presence of contextual cues, selective mapping $Q(s,a,C)$ representing stimulus-cue-response associations. Each selective mapping is learned through the standard temporal difference RL rule described above with learning rates α_S and α_C. In general, behavioral policy $P(a_t|s_t,C_t)$ then forms a mixture of ε-softmax with inverse temperature β and β' (and Q-values normalized over actions):

$$P\left(a_t \middle| s_t, C_t\right) = (1-\varepsilon)\left[(1-\omega)\frac{\exp \beta Q(s_t,a_t)}{\sum_{a=1\ldots n_a} \exp \beta Q(s_t,a)} + \omega \frac{\exp \beta' Q(s_t,a_t,C_t)}{\sum_{a=1\ldots n_a} \exp \beta' Q(s_t,a,C_t)}\right] + \frac{\varepsilon}{n_a},$$

where ω is the mixture rate. In experiment 1 including no contextual cues, mixture rate ω was set to 0 and parameter α_C, β' were removed from the fitting: the model thus included only three free parameters β, ε, α_S. In experiment 2, all parameters were included: β, β', ε, α_S, α_C, and ω.

3-Data analyses

Proportions of responses. In every behavioral session, the first episode was systematically factored out from data analyses. Because the mappings between stimuli and correct responses used in two successive episodes were fully incongruent, subjects then produced three types of responses: (1) *correct* responses in the ongoing episode (one response to every stimulus); (2) *perseverative* responses corresponding to the correct responses in the preceding episode (one response to every stimulus); (3) *exploratory* responses corresponding to neither correct nor perseverative responses (two responses to every stimulus). We report only the proportions of correct and exploratory responses, because the proportions of correct, perseverative and exploratory responses sum up to 1.

Mutual dependence between successive decisions. The mutual dependence between two successive decisions was computed as the (mutual) information $I(t,t+1)$ that the response outcome in trial t provide for selecting a correct response in trial $t+1$:

$$I(t,t+1) = \sum_{a_t \in \{0,1\}} \sum_{a_{t+1} \in \{0,1\}} P(a_t, a_{t+1}) \log \frac{P(a_t, a_{t+1})}{P(a_t) P(a_{t+1})},$$

where a_t=1 and a_t=0 denote correct and incorrect responses, respectively. $P(.)$ denotes observed frequencies of actual responses in trial t and $t+1$ over episodes (t=0 refers to episode onsets).

Model fitting. Models were simulated using binary inputs for digit stimuli and color cues**.** Stimuli were modeled as binary indicators coding for digits only. Contextual cues were modeled as binary indicators coding for either color-digit pairs or colors only (because subjects were not informed about the meaning of color cues). The same results were obtained in both cases.

For every model, we used three fitting criteria: (1) ordinary least squares (LS) minimizing the residual square distance between observed frequencies and predicted probabilities of correct responses over sessions. Predicted probabilities were computed in every trial according to the preceding responses produced by subjects; (2) log-likelihood (LLH) maximizing the (log-) likelihood of observing actual subjects' responses in every trial given the model and the preceding responses produced by subjects; (3) Bayesian information criterion (BIC) altering LLHs according to the number of model free parameters. The BIC penalizes models with larger number of free parameters. Fittings were performed by combining grid search and gradient descents with multiple starting points using the MATLAB optimization toolbox. Model fits were compared according to these three fitting criteria. Lower LSs, larger LLHs and lower BICs indicate better fits. As shown in Results (**Figs. 2 & 8**), the PROBE model was the best fitting model regardless of fitting criteria.

Statistical analyses. In agreement with standard statistical analyses, we report parametric test values (T-tests and F-tests) for subjects' performances, whenever the number of subjects was larger than 20. Otherwise, we report non-parametric test values as mentioned in the text.

For variations of model parameters across groups, we report non-parametric statistical tests as mentioned in the text regardless of subjects' number, because model parameters are bounded, sometimes discrete (bounds N) and the underlying distribution may depart from the normal distribution.

4- Post-tests

Experiment 1. Immediately after the recurrent session, we administered a post-test to subjects measuring their ability to retrieve the three mappings between stimuli and correct responses. The three stimuli used during the session were shown on the screen within arrays representing the four response keys (also shown as positive feedbacks, see above). 6 combinations were shown to each subject: three showing the three mappings that she/he encountered in the recurrent session (i.e. each stimulus was placed on the corresponding correct keys) and three showing those encountered by another subject in her/his own recurrent session. Subjects had to rate each combination from 0 to 4 as follows:

0: I am certain this combination was not valid during the session
1: I think this combination was not valid during the session
2: I don't know whether this combination was valid during the session
3: I think this combination was valid during the session
4: I am certain this combination was valid during the session.

We then computed a *discrimination* score for every subject as the maximal rating of no-encountered combinations subtracted from the minimal rating of encountered combinations. Positive discrimination scores mean that all encountered combinations were rated higher than other combinations. 13 subjects exhibited positive discrimination scores and were referred to as *exploiting* subjects. The 9 remaining subjects were referred to as *exploring* subjects. We found no significant correlations between discrimination scores and age, gender or session order (all Ps > 0.05).

Experiment 2. As in the first experiment, we administered a post-test to subjects immediately after the second behavioral session. The post-test measured subjects' ability to retrieve the mapping between stimuli and correct responses as well as associated contextual cues they encountered in the session. Because there were many potential combinations mixing contextual cues and mappings, we simply asked subjects to write down the mappings and associated cues they could remember (without any feedbacks). Similar to the first experiment, three groups of subjects were then determined as follows:

*S*ubjects who wrote down the three recurrent mappings encountered in the session formed the *exploiting* group (34 subjects). The remaining ones formed the *exploring* group (15 subjects). Within the exploiting group, those who further wrote down the two contextual cues associated with the mappings used in control episodes were referred to as *context-exploiting* subjects (19 subjects). The remaining ones were referred to as *outcome-exploiting* subjects (15 subjects). Again, no significant correlations were found between groups and age and gender (all Ps>0.05).

5. Comments on model fits.

In the first experiment, subjects produced significantly more exploratory responses on trial 5 following episode onsets than model predictions (see Fig. 3). In trial 5, exploratory responses peaked so that *on average*, subjects had quit the ongoing task-set in the preceding trial. On these trials, moreover, correct responses were appropriately predicted. Consequently, the discrepancy implies that subjects performed less *perseverative* responses than the models, so that subjects exhibited an *inhibition of return bias* preventing subjects from reusing the

mappings they just quit[see e.g. 4]. This bias was not implemented in the models for simplicity. We may implement the bias by penalizing the reliability of task-sets that subjects just quit. In the PROBE model, the bias would decrease the weight of the corresponding mappings in the mixture used for creating task-sets. The bias would have an observable influence on behavior especially when the total number of behavioral strategies stored in long-term memory and associated with the same context remains small. This number is the lowest in recurrent episodes and exploiting subjects. Consistently, the discrepancy is observable only in these cases (see Fig. 6). Note that the effect is not observed in experiment 2, because contextual cues changed at episode onsets.

In the control experiment including four recurrent action sets (Fig. 4S), subjects performed on average as in the open condition, while according to the PROBE model, the monitoring capacity was on average equal to three task sets. To understand this point, note first that the pseudo-randomization was such that about 50% of episodes associated with one action set followed the successive occurrences of the three other action sets. With a monitoring capacity limited to three task sets, consequently, the corresponding task set is removed from the monitoring buffer at onsets of such episodes, which become equivalent to open episodes. In this control experiment, moreover, post-tests revealed that as in Experiment 1, 1/3 of subjects were classified as exploring subjects and behaved similarly in recurrent and open episodes. Consequently, the overall performance averaged over episodes and subjects shown in Fig. 4S appears similar to that found in open episodes from experiment 1. As expected, however, the performance of exploiting subjects significantly improved in episodes associated with one action set and preceded by the successive occurrences of only one or two other action sets compared to those preceded by the successive occurrences of three other action sets (increases of correct responses: $T=1.9$, $p<0.06$; decreases of exploratory responses: $T=2.4$, $p<0.016$).

References

1. Ferguson TS (1973) A Bayesian analysis of some non-parametric problems. Annals of Statistics 1: 209-230.
2. Geman S, Geman D (1984) Stochastic relaxation, Gibbs distributions, and the Bayesian restoration of images. IEEE Transactions of Pattern Analysis and Machine Intelligence 6: 721-741.
3. Daw ND, Courville A (2007) The pigeon as particle filter. Advances in Neural Information Processing Systems 20.
4. Dreher J-C, Berman KF (2002) Fractionating the neural substrate of cognitive control processes. Proc Natl Acad Sci USA. pp. 14595-14600.
